# Inhibition of NEK2 Promotes Chemosensitivity and Reduces KSHV-positive Primary Effusion Lymphoma Burden

**DOI:** 10.1158/2767-9764.CRC-23-0430

**Published:** 2024-04-09

**Authors:** Maria C. White, Jason P. Wong, Blossom Damania

**Affiliations:** 1 Lineberger Comprehensive Cancer Center, the University of North Carolina at Chapel Hill, Chapel Hill, North Carolina.; 2 Department of Microbiology and Immunology, the University of North Carolina at Chapel Hill, Chapel Hill, North Carolina.

## Abstract

Non–Hodgkin lymphoma (NHL) is a common cancer in both men and women and represents a significant cancer burden worldwide. Primary effusion lymphoma (PEL) is a subtype of NHL infected with Kaposi sarcoma–associated herpesvirus (KSHV). PEL is an aggressive and lethal cancer with no current standard of care, owing largely to its propensity to develop resistance to current chemotherapeutic regimens. Here, we report a reliance of KSHV-positive PEL on the mitotic kinase, NEK2, for survival. Inhibition of NEK2 with the inhibitor, JH295, resulted in caspase 3–mediated apoptotic cell death of PEL. Furthermore, NEK2 inhibition significantly prolonged survival and reduced tumor burden in a PEL mouse model. We also demonstrate that the ABC transporter proteins, MDR1 and MRP, are most active in PEL and that inhibition of NEK2 in PEL reduced the expression and activity of these ABC transporter proteins, which are known to mediate drug resistance in cancer. Finally, we report that JH295 treatment sensitized lymphomas to other chemotherapeutic agents such as rapamycin, resulting in enhanced cancer cell death. Overall, these data offer important insight into the mechanisms underlying PEL survival and drug resistance, and suggest that NEK2 is a viable therapeutic target for PEL.

**Significance:**

The mitotic kinase, NEK2, is important for the survival of KSHV-positive PEL. NEK2 inhibition resulted in PEL apoptosis and reduced tumor burden in a mouse model. NEK2 inhibition also reduced drug resistance.

## Introduction

Non–Hodgkin lymphoma (NHL) encompasses a diverse array of lymphomas with differing etiologies and is among the top ten most common cancers diagnosed in both men and women (1). Viral infection is associated with numerous different NHL, and one of these lymphomas, primary effusion lymphoma (PEL), occurs in individuals infected with Kaposi sarcoma–associated herpesvirus (KSHV). In PEL, KSHV-infected malignant B-cell effusions fill the body cavities surrounding the heart, lungs, and abdomen (2). The prognosis for PEL is extremely poor, with a median overall survival of less than one year (3). In addition, there is no standard of care, and few treatment options currently exist for PEL, highlighting the dire need for novel therapeutic targets for this aggressive cancer (4). Furthermore, PEL is frequently refractory to chemotherapeutics currently in clinical use and can quickly develop resistance toward these drugs, further complicating treatment of this malignancy (5). Therefore, it is of great interest to develop alternative treatment strategies for PEL that not only significantly reduce tumor burden but also combat the issue of drug resistance to chemotherapy.

We performed a kinome screen on viral and nonviral NHL and identified cellular kinases that were upregulated in NHL relative to B cells isolated from healthy donors (6). One of the kinases upregulated in PEL compared with primary B cells was NIMA related kinase 2 (NEK2). NEK2 is a serine/threonine kinase involved in mitotic processes such as centrosomal separation, kinetochore attachment, and chromosomal alignment (7–10). Overexpression of NEK2 in normal fibroblasts was shown to increase cellular proliferation (11), suggesting that aberrant NEK2 expression can lead to uncontrolled cell division and cancer development. Importantly, NEK2 depletion had no effect on the growth of normal fibroblasts, but reduced the growth of malignant cells (12). These data suggest that, although both transformed cells and normal cells express NEK2, transformed cells may possess a unique dependency on NEK2 for survival. Indeed, studies have reported that other tumors display NEK2 expression (11, 13–15); however, the mechanisms by which NEK2 controls these processes in cancer remain poorly characterized, and its role in viral lymphomas, including PEL, is unknown.

Here, we used genetic and pharmacologic approaches to test the importance of NEK2 in PEL survival. We show that PEL viability is significantly decreased when cellular NEK2 expression is depleted and/or when NEK2 activity is inhibited. In the absence of intact NEK2 signaling, PEL cells undergo caspase 3–mediated apoptosis and cell cycle arrest. Importantly, NEK2 inhibition does not affect the viability of healthy cells, including primary B cells that are actively proliferating. In addition, we found that NEK2 inhibition decreases the expression of cellular proteins involved in cell growth. Furthermore, we identify the multidrug resistance proteins, multidrug resistance 1 (MDR1) and multidrug resistance-associated protein (MRP), as the drug transporter proteins most active in PEL, and show that NEK2 inhibition decreases the expression and activity of these transporter proteins, which are known to be involved in efflux of chemotherapeutic drugs from cells. Using a PEL xenograft mouse model, we found that NEK2 inhibition significantly prolongs host survival and decreases PEL burden as measured by ascites volume, tumor weight, and *in vivo* imaging, with no detectable liver toxicity. Finally, we demonstrate that NEK2 inhibition can sensitize lymphomas to chemotherapy, resulting in a synergistic effect on lymphoma cell death. Taken together, these data may offer a new therapeutic approach for patients with PEL, and provide insight into the mechanisms of PEL proliferation and drug resistance.

## Materials and Methods

### Cell Lines, Cell Culture, and Inhibitors

PEL cell lines BCBL1 (RRID:CVCL_0165), BC1 (RRID:CVCL_1079), and JSC1 (RRID:CVCL_3728) were grown in RPMI1640 medium (Corning, 10–040-CV) supplemented with 10% FBS, 1% penicillin–streptomycin, 1% l-glutamine, 0.075% sodium bicarbonate (Gibco, 25080–094), and 0.05 mmol/L β-mercaptoethanol (Gibco, 21985–023). 293FT (RRID:CVCL_6911) cells were grown in DMEM supplemented with 10% FBS, 1% penicillin–streptomycin, 1% l-glutamine, and 500 µg/mL geneticin (Gibco, 10131–035). Trex-RTA BCBL1-luc cells were grown in RPMI1640 medium supplemented with 10% Tet-free FBS, 1% penicillin–streptomycin, 1% l-glutamine, 20 µg/mL hygromycin B (Corning, 30–240-CR), and 1.25 µg/mL puromycin (Corning, 61385RA). Peripheral blood mononuclear cells (PBMC; commercially obtained) were maintained in RPMI1640 medium (Corning, 10–040-CV) supplemented with 10% FBS, 1% penicillin–streptomycin, and 1% l-glutamine. Cell lines were previously authenticated by high-throughput sequencing and short tandem repeat profiling (ATCC). PBMCs were either commercially aquired (STEMCELL or StemExpress) or had previously been isolated in-house from commercially aquired source leukocytes (Gulf Coast Regional Blood Center) and stored in liquid nitrogen. PEL cells and Trex-RTA BCBL1 cells were (provided by Jae Jung, Cleveland Clinic) and 293FT cells (purchased from Thermo Fisher Scientific) were used in experiments until 20–25 passages after thawing. Trex-RTA BCBL1-luc cells (provided by Dirk Dittmer, University of North Carolina at Chapel Hill, Chapel Hill, NC) were used for mouse experiments within 3 weeks of thawing. PBMCs were used in experiments immediately after thawing and were not passaged. PEL cell stocks were tested for *Mycoplasma* (LookOut Mycoplasma PCR Detection Kit, Sigma, MP0035) before initiation of experiments.

JH295 was purchased from Tocris (Bio-Techne, 4322), reconstituted to 50 mmol/L in dimethyl sulfoxide (DMSO; Sigma, D2650), and stored at −20°C protected from light. NCL-00017509 was purchased from Tocris (Bio-Techne, 5150), reconstituted to 100 mmol/L in DMSO, and stored at −20°C protected from light. A-1155463 was purchased from Selleckchem (S7800), reconstituted to 50 mmol/L in DMSO, and stored at −80°C. Verapamil hydrochloride was purchased from MedChemExpress (HY-A0064), reconstituted to 50 mmol/L in DMSO, and stored at −80°C protected from light. MK-571 sodium was purchased from MedChemExpress (HY-19989A), reconstituted to 50 mmol/L in DMSO, and stored at −80°C protected from light. Novobiocin was purchased from MedChemExpress (HY-B0425A), reconstituted to 45 mmol/L in DMSO, and stored at −80°C protected from light. T-1101 tosylate was purchased from MedChemExpress (HY-120356A), reconstituted to 10 mmol/L in DMSO, and stored at −80°C protected from light. Vinblastine (22 mmol/L in DMSO) was purchased from Sigma-Aldrich (90301) and stored at 4°C protected from light. Rapamycin (Sirolimus) was purchased from Selleckchem (S1039), reconstituted to 20 mmol/L in DMSO, and stored at −20°C.

For *in vivo* experiments, sterile 100% DMSO (20 µL injection volume) was used as the vehicle control. JH295 was used at a concentration of 15 mg/kg; assuming animal weight of approximately 0.02 kg, each mouse received 0.3 mg JH295. JH295 was dissolved in 100% DMSO to a final concentration of 15 mg/mL (20 µL injection volume). Drugs were prepared fresh weekly for injection.

### Short Hairpin RNA Knockdowns

For depleting NEK2 in PEL cell lines, Sigma MISSION short hairpin RNAs (shRNA) were used. pLKO.1-puromycin control (Sigma, SHC002) and the following NEK2 shRNAs in the pLKO.1 backbone (Sigma, SHCLNG-NM_002497) were used: TRCN0000000948 (shRNA #2) and TRCN0000195573 (shRNA #4). Lentivirus particles containing one of each of these three puromycin plasmids were then generated in 293FT cells using the ViraPower Lentiviral Packaging Mix (Thermo Fisher Scientific, K497500) according to the manufacturer's instructions. Lentiviruses were harvested, cleared of cellular debris, and either used immediately or frozen at −80°C for later use. PEL cells were seeded in 6-well plates at a density of 1 million cells per well in the absence of serum and antibiotics. Immediately postseeding, cells were spinfected with 1 mL lentivirus/well via centrifugation at 3,500 × *g* for 1.5 hours at 30°C in the presence of polybrene (10 µg/mL; Sigma, TR-1003). Cells were then immediately incubated at 37°C. The next day, the media was replaced with complete media containing 1 µg/mL puromycin (Corning, 61385RA) and cells were transferred to T25 flasks. After 72 hours of puromycin selection, PEL cells were harvested and either used in Western blotting for NEK2 expression analysis or seeded for cell viability assays (below). Puromycin selection was maintained throughout the cell viability experiments (BCBL1 500 ng/mL, BC1 250 ng/mL, JSC1 500 ng/mL).

### siRNA Knockdowns

ON-TARGETplus Human NEK2 SMARTpool siRNA (Dharmacon, 4751) was used to deplete NEK2 protein from PEL cells (J-004090–18: GGAUCUGGCUAGUGUAAUU, J-004090–19: GCAGACAGAUCCUGGGCAU, J-004090–20: GGCAAUACUUAGAUGAAGA, and J-004090–21: GCUAGAAUAUUAAACCAUG). The ON-TARGETplus control pool siRNA (Dharmacon, D-001810–10–20) was used as the nontargeting control (NTC). The siRNAs were reconstituted in 5x siRNA buffer (Dharmacon, B-002000-UB-100) in water for a final concentration of 1x. The Amaxa Cell Line Nucleofector Kit (Lonza, VCA-1003) was used for nucleofection of PEL cells following the manufacturer's instructions. Two micrograms of siRNA was used per sample, each containing 1 × 10^6^ PEL cells in log phase. Transfected cells were incubated at 37°C for 48 hours before being used for protein or RNA extraction.

### Cell Viability Assays

For IC_50_ assays, PEL cells were seeded at a density of 1 × 10^4^ cells/well in triplicate in white 96-well plates (Greiner, 655083) in 100 µL volume. Immediately prior to plating, cells were mock treated (0.1% DMSO) or treated with 1:2 serial dilutions of JH295 in DMSO, starting at 2 µmol/L. One plate was seeded per time point assayed. Plates were incubated at 37°C. At the indicated time points, a CellTiter-Glo Luminescent Cell Viability assay (Promega, G7573) was performed according to the manufacturer's instructions. Twenty-five microliters of prepared reagent was added to each well. Luminescence was then recorded using a CLARIOstar Plus Plate Reader (BMG Labtech). IC_50_ values were calculated using IC_50_.tk software.

For Trypan blue and cell death assays, PEL cells were seeded at a density of 2.5 × 10^5^ cells/mL in duplicate and treated with or without JH295 for 48 hours. Cells were then harvested, resuspended in PBS, and percent dead cells, as well as live cells per mL, were quantified using Trypan blue exclusion staining and a Countess II FL automated cell counter (Life Technologies).

For measuring cell viability over time, PEL cells or PBMCs were seeded at a density of 1 × 10^4^ cells/well or 1 × 10^5^ cells/well, respectively, in triplicate in white 96-well plates (Greiner, 655083) in 100 µL volume. Immediately prior to plating, cells were mock treated (0.1% DMSO) or treated with various drugs at the indicated concentrations. One plate was seeded per time point assayed. A 0-hour plate was included for luminescence data normalization. Plates were incubated at 37°C. At the indicated time points, CellTiter-Glo Luminescent Cell Viability assays (Promega, G7573) were performed according to the manufacturer's instructions. Twenty-five microliters of prepared reagent was added to each well. For the 0-hour time points, plates were incubated postseeding at 37°C for approximate 1 hour and then the assay was performed. Luminescence was recorded using a CLARIOstar Plus Plate Reader (BMG Labtech). The same gain was used across all time points.

### Primary B-Cell Assays

Primary B cells were isolated from commercially acquired PBMCs (STEMCELL or StemExpress) or frozen PBMCs previously isolated in-house from commercially aquired source leukocytes (Gulf Coast Regional Blood Center) using the EasySep Human B Cell Isolation Kit (STEMCELL, 17954) per the manufacturer's instructions. PBMCs were washed and treated with DNase I solution (Invitrogen, 18047–019) for 15 minutes at room temperature prior to B-cell isolation. Isolation buffer was made using DPBS (Corning, 21–031-CV) supplemented with 2% FBS and 1 mmol/L EDTA (Corning, 46–034-Cl). Isolated B cells were used immediately and maintained in RPMI1640 medium supplemented with 10% FBS, 1% penicillin–streptomycin, 1% l-glutamine, 1x MEM NEAA (Gibco, 11140–050), 55 µmol/L β-mercaptoethanol (Gibco, 21985–023), 10 mmol/L HEPES buffer (Corning, 25–060-Cl), and 1 mmol/L sodium pyruvate (Gibco, 11360–070). Purity of isolated B cells was verified by flow cytometry analysis (MACSQuant VYB, Miltenyi Biotec) on PBMCs preisolation and primary B cells postisolation. B-cell populations of >95% purity were used. Cells were resuspended in staining buffer (BioLegend, 420201) with or without antibodies [Alexa Fluor 594 Anti-Human CD3 antibody, BioLegend (300446); Pacific Blue Anti-Human CD20 antibody, BioLegend (302319)] and incubated on ice for 40 minutes protected from light. Cells were then washed two times with 500 µL staining buffer, resuspended in 500 µL staining buffer, and analyzed via flow cytometry. For cell viability assays, 1 × 10^5^ primary B cells per well were plated in white 96-well plates (Greiner, 655083) in 100 µL volume. Each treatment was performed at least three independent times in triplicate (with the exception of one biological replicate in duplicate) using unique cell donors. For LPS stimulation or mock stimulation, cells were treated for 4 hours at 37°C with 10 µg/mL LPS (Sigma-Aldrich, L4005) or PBS, respectively. Four hours later, cells were then mock treated (0.1% DMSO) or treated with various concentrations of JH295 and incubated at 37°C for 48 hours. CellTiter-Glo Luminescent Cell Viability assays (Promega, G7573) were then performed as described above.

### Western Blotting

**FIGURE 1:**
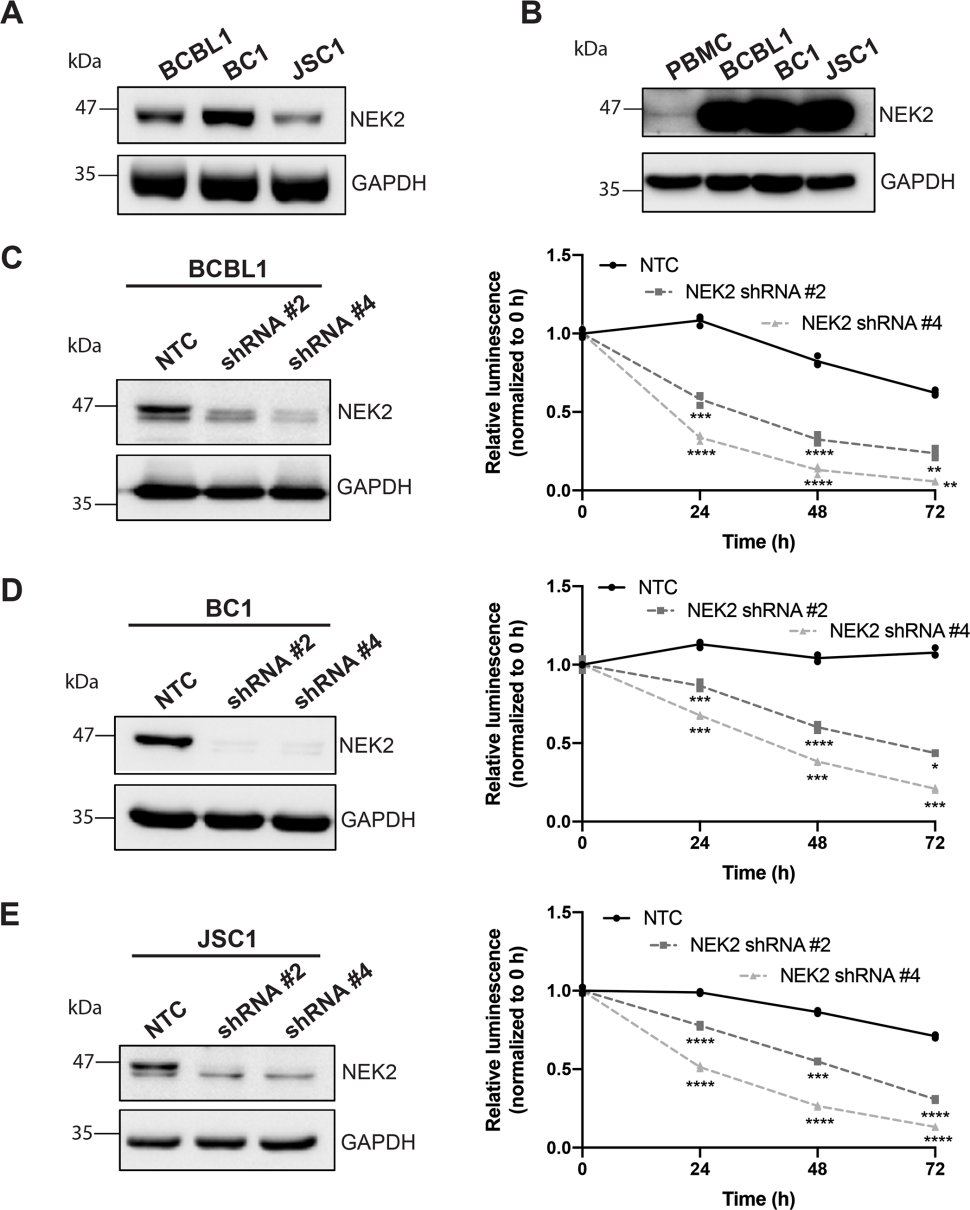
NEK2 depletion decreases PEL survival. **A,** Western blot analysis for NEK2 expression in PEL cells. **B,** Western blot analysis for NEK2 expression in normal PBMCs alongside PEL cells. **C–D,** Western blots for shRNA-mediated knockdown of NEK2 expression in PEL cells and subsequent cell viability assays measuring PEL survival over time after treatment with two different NEK2 shRNAs (dotted lines) or nontemplate control (NTC, solid black line). Data are representative of three independent biological replicates, each performed in triplicate. Viability data were normalized to 0-hour luminescence values for each treatment, fitted using nonlinear regression, and analyzed using two-way ANOVA with Dunnett multiple comparisons. ****, *P* < 0.0001. **C,** ***P = 0.0001; **, *P* < 0.0005. **D,** ***, *P* < 0.0005; *, *P* = 0.0009, **E,** ***, *P* = 0.0003. The BCBL1 loading control blot in C is the same as in Fig. 3E.

After treatment with or without JH295 for 48 hours, cells were harvested and lysed on ice for 25 minutes using 0.1% NP40 lysis buffer [0.1% NP40, 50 mmol/L Tris HCl pH 8, 150 mmol/L NaCl, 30 mmol/L β-glycerophosphate, 50 mmol/L NaF, 1 mmol/L Na_3_VO_4_, 1 protease tablet (Roche, 11873580001), and molecular grade water (Corning, 46000-CM)]. Lysates were cleared of debris by centrifugation at 15,800 × *g* at 4°C for 10 minutes. Cleared lysates were collected and quantified via Bradford Assay (Bio-Rad Protein Assay Dye, 5000006). Lysates were combined with 5x urea buffer containing β-mercaptoethanol and boiled for 5 minutes at 95°C. For each sample, 50 µg protein lysate was loaded and proteins were resolved via SDS-PAGE and transferred onto 0.45-µm nitrocellulose membranes. Membranes were blocked using either 5% nonfat dry milk (Apex Chemicals and Reagents, 20–241) or 5% BSA (Sigma-Aldrich, A7906), with the exception of the blot for NEK2 expression in PBMCs alongside PEL, which was blocked using 1% nonfat dry milk. Antibodies were purchased from either Cell Signaling Technology (CST) or Santa Cruz Biotechnology. Primary antibodies: NEK2 (1:1,000 or 1:250, Santa Cruz Biotechnology, 55601; RRID:AB_1126558), GAPDH (1:2,500, Santa Cruz Biotechnology, 47724; RRID:AB_627678), caspase 3 (CST, 1:1,000, 9665S; RRID:AB_2069872), PARP (1:1,000, CST, 9542S; RRID:AB_2160739), MDR1 (1:1,000, CST, 13342S; RRID:AB_2631176), MRP1 (1:1,000, CST, 72202S; RRID:AB_2799816), BCRP (1:1,000, CST, 42078S; RRID:AB_2799211), total β-catenin (1:1,000, CST, 8480S; RRID:AB_11127855), phosphorylated β-catenin S33/37/T41 (1:1,000, CST, 9561S; RRID:AB_331729), and Bcl-xL (1:1,000, CST, 2764S; RRID:AB_2228008). The KSHV ORF45 antibody (RRID:AB_10999794) was purchased from Thermo Fisher Scientific (MA5–14769) and diluted 1:1,000. The following secondary antibodies were used: anti-rabbit IgG-HRP (1:2,000, CST, 7074S; RRID:AB_2099233) and anti-mouse IgG-HRP [1:2,000 or 1:1,000 (Fig. 1B), CST, 7076S; RRID:AB_330924]. Blots were developed using either Clarity Western ECL Substrate (Bio-Rad, 1705061) or Amersham ECL Prime (Cytiva, RPN2232) and imaged using a Bio-Rad ChemiDoc Touch Imaging System. Images were exported and viewed using Image Lab software.

### RT-qPCR

RNA was isolated from cell pellets using the RNeasy Plus Mini Kit (QIAGEN, 74134) following the manufacturer's instructions. A genomic DNA elimination step was included. Isolated RNA was eluted in 30 µL molecular grade water and nanodropped to determine RNA concentration. One microgram of RNA per sample was used for cDNA synthesis (Bio-Rad iScript gDNA Clear cDNA Synthesis Kit, 1725035) following the manufacturer's instructions with an additional gDNA elimination step prior to reverse transcription. cDNA was diluted 1:5 in water and qPCR was performed using SensiFAST SYBR Lo-Rox (Bioline, BIO-94020) and a QuantStudio 6 Flex Real-Time PCR system (Applied Biosciences). Forward and reverse primers were used at a final concentration of 250 nmol/L each. Gene expression was normalized to actin and fold changes were calculated using the 2^−ΔΔ^^*C*_t_^ method. RTA forward (F): GAACTGAAGGCCCAACTCTAC; RTA reverse (R): CACACATCTTCCACCACTCTATT; vIL6 F: CGGTTCACTGCTGGTATCTG; vIL6 R: CAGTATCGTTGATGGCTGGT; ORF57 F: TGGACATTATGAAGGGCATCCTA; ORF57 R: CGGGTTCGGACAATTGCT; K8.1 F: AAAGCGTCCAGGCCACCACAGA; K8.1 R: GGCAGAAAATGGCACACGGTTAC; Actin F: AAGACCTGTACGCCAACACA; Actin R: AGTACTTGCGCTCAGGAGGA.

### Caspase 3 Assays

Caspase 3 activity assays were performed using the ApoAlert Caspase 3 Fluorescence Assay Kit (Takara Bio, 630215) following the manufacturer's instructions. One-hundred microliters of lysis buffer was used as the background control.

### Apoptosis/Annexin V Assays

The percentage of annexin V–positive cells was determined using the FITC Annexin V Apoptosis Detection Kit with PI (BioLegend, 640914) following the manufacturer's instructions. Manufacturer's recommended volumes of FITC Annexin V and propidium iodide (PI) were halved. Subsequent data analysis included both the FITC^+^ PI^−^ and the FITC^+^ PI^+^ populations.

### Cell-Cycle Assays

PEL cells were treated with or without JH295 for 24 hours and were then harvested and washed 2× with 10 mL ice-cold PBS. One to two million cells per sample were then resuspended in 500 µL ice-cold PBS and fixed/permeabalized using ice-cold 70% ethanol added gradually while vortexing gently. Cells were fixed on ice in the refrigerator for 2–3 hours. Cells were then spun at 500 × *g* for 5 minutes at 4°C and supernatant was gently removed. Cells were then washed two times with 5 mL PBS at 500 × *g* for 5 minutes at 4°C. Cells were resuspended in 500 µL staining solution and incubated for 30 minutes at 37°C protected from light. Staining solution consisted of 10 µg/mL RNase A (Thermo Fisher Scientific, EN0531) to ensure DNA-only staining, PI solution (BioLegend, 421301), and cell staining buffer (BioLegend, 420201). Cells were then placed on ice and analyzed via flow cytometry (MACSQuant VYB, Miltenyi Biotec) on a linear scale using the PI (B2) channel.

### Multidrug Resistance Assays

For the Multidrug Resistance Direct Dye Efflux Assay, a kit of the same name (Sigma, ECM910) was used. The protocol for the dye, DiOC_2_(3), was used following the manufacturer's instructions. Cells were kept on ice unless actively effluxing dye at 37°C. A verified 37°C water bath was used for the efflux step. Cells were protected from light throughout the duration of the assay.

To calculate the multidrug resistance activity factor (MAF), the following equation was used for each sample: 100 – [100 × ((mean fluorescence intensity_sample_ – mean fluorescence intensity_DMSO control_)/mean fluorescence intensity_sample_)].

For the eFLUXX-ID Green Multidrug Resistance Assay, a kit of the same name (Enzo, ENZ-51029-K100) was used. Reagents were prepared according to the manufacturer's instructions using anhydrous DMSO (Biotium, 90082). Cells were grown and assayed in complete phenol red–free RPMI medium (Gibco, 11835–030). Media containing anhydrous DMSO was used for control cells that did not receive green dye and/or inhibitors. The assay was performed according to the manufacturer's instructions. Cells were loaded with dye for 10 minutes at 37°C in a verified 37°C water bath. The efflux step was allowed to proceed for 1 hour at 37°C in a verified 37°C water bath. The green dye becomes fluorescent only when inside cells and cleaved by esterases. As these esterases are active at 37°C, a sample loaded with dye but continuously kept on ice served as a negative control. Cells loaded with dye and incubated at 37°C but treated with all three ABC transporter inhibitors to prevent dye efflux was used as a positive control.

### Mouse Experiments

Animal experiments were performed by the Preclinical Research Unit (PRU) at the University of North Carolina at Chapel Hill (Chapel Hill, NC) and were approved by the Institutional Animal Care and Use Committee (IACUC) at the University of North Carolina at Chapel Hill (Chapel Hill, NC). Female NOD/SCID/gamma (NSG) mice (strain name NOD.Cg-Prkdc^scid^Il2rg^tm1Wjl^/SzJ; RRID:BCBC_1262), 6–8 weeks of age and weighing approximately 20 g, were purchased from The Jackson Laboratory by the PRU. Mice were maintained under pathogen-free conditions and had free access to food and water. Mice were injected intraperitoneally with 70,000–100,000 Trex-RTA BCBL1-luciferase PEL cells in sterile PBS in the lower right abdominal quadrant. Injection of this cell number results in PEL establishment in NSG mice detectable by live imaging, enabled by the luciferase construct present in the Trex-RTA BCBL1-luciferase PEL cells (6). Three days post-PEL cell injection, mice were randomized into treatment groups according to tumor burden (determined by imaging) and were treated three times per week (Monday, Wednesday, Friday) with 20 µL 100% sterile DMSO (vehicle control) or 20 µL 15 mg/kg JH295 intraperitoneally until their body score index or tumor burden reached humane IACUC limits (survival experiments) or for 23 days (direct tumor burden comparison experiment). Mice were imaged pre- and postrandomization and every Monday thereafter using the Xenogen IVIS-Lumina system (Caliper Life Sciences) following intraperitoneal injection with 10 µL per gram body weight of luciferin (PerkinElmer). For the survival experiments, *N* = 22 for each treatment group is pooled from three independent biological replicates (*n* = 5, *n* = 8, and *n* = 9 per treatment group).

### Statistical Analyses

Sample sizes were determined using G*Power software for 85%–95% power to detect a two-fold difference between groups using a 0.05 α value and Wilcoxon–Mann–Whitney test. *t* test was used to compare two variables; ANOVA was used to compare three or more variables. Specific tests for each data set are indicated in the figure legends. The chosen *post hoc* multiple comparisons tests were performed as recommended by the analysis software. *P* values <0.05 were considered significant. All data were analyzed using GraphPad Prism 9. All flow cytometry data were processed using FlowJo v10.8.0 software. Unless otherwise stated, all experiments were performed three independent times on different days.

### Data Availability

The data generated in this study are available upon request from the corresponding author.

## Results

### NEK2 Expression Promotes PEL Viability

We first confirmed at the protein level that the PEL cell lines BCBL1, BC1, and JSC1 express NEK2 (Fig. 1A) and that NEK2 protein levels are elevated in PEL compared with normal peripheral blood mononuclear cells (PBMCs; Fig. 1B). To assess the role of NEK2 in PEL survival, we transduced PEL cells with lentiviruses expressing one of two different shRNAs against NEK2. Depletion of NEK2 protein in all the cell lines was confirmed (Fig. 1C–E) and the viability of the PEL cells with depleted NEK2 was compared with that of the nontemplate control (NTC) cells over time (Fig. 1C–E). Results showed that, in all cell lines tested, PEL viability was significantly reduced in the cells depleted for NEK2 compared with the control cells that fully expressed NEK2 (Fig. 1C–E), suggesting that NEK2 expression is required for PEL survival.

### Inhibition of NEK2 Results in Death of PEL Cells but has no Impact on Normal B Cells

**FIGURE 2:**
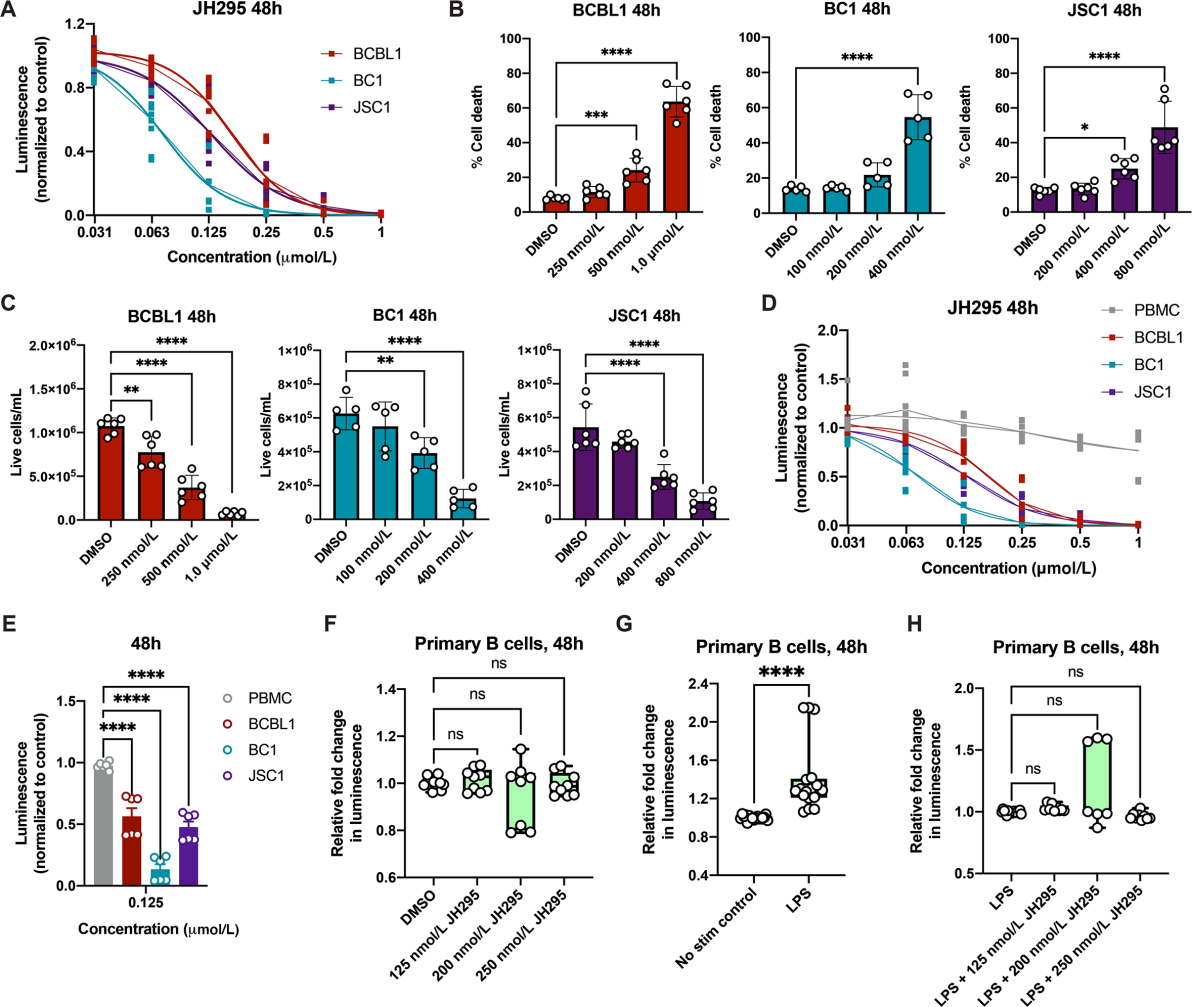
JH295 treatment results in death of PEL cells but has no impact on normal B cells. **A,** IC_50_ curves of PEL cell lines treated with a range of JH295 concentrations and normalized to the DMSO control values. Curves were fitted using nonlinear regression and are connected by means. Data are four independent biological replicates, each performed in triplicate. See also Supplementary Table S1. **B,** Quantification of PEL cell death via Trypan blue staining following JH295 treatment. Individual data points from three independent biological replicates are plotted as mean ± SD and analyzed using one-way ANOVA with Dunnett multiple comparisons. ****, *P* < 0.0001; **, *P* = 0.0003; *, *P* = 0.04. **C,** PEL cell viability via Trypan blue count following mock or JH295 treatment. Individual data points from three independent biological replicates are plotted as mean ± SD and analyzed using one-way ANOVA with Dunnett multiple comparisons. ****, *P* < 0.0001; **, *P* < 0.007. **D,** IC_50_ curve of bulk PBMCs treated with JH295 and normalized to the DMSO control values, overlayed on the PEL data from A. Curves were fitted using nonlinear regression and are connected by means. Data are three individual biological replicates, each performed in triplicate, using two unique PBMC donors. **E,** Cell viability of PBMCs versus PEL cells treated with JH295, each normalized to the corresponding DMSO control values. Individual data points from two independent biological replicates using two unique PBMC donors and performed in triplicate are plotted as mean + SEM and analyzed using one-way ANOVA with Dunnett multiple comparisons. ****, *P* < 0.0001. **F,** Cell viability of purified primary B cells treated with either DMSO or JH295. Data representing fold change in luminescence relative to the DMSO control, are plotted as individual data points of three to six independent biological replicates using three to six unique cell donors, and analyzed using unpaired two-tailed *t* test. ns, not significant. See also Supplementary Fig. S1. **G,** Cell viability of purified primary B cells following mock or LPS stimulation. Data are fold change in luminescence relative to mock-stimulated cells, are plotted as individual data points of three to six independent biological replicates using three-six unique cell donors, and analyzed using unpaired two-tailed *t* test. **, *P* = 0.0031. **H,** Cell viability of stimulated purified primary B cells treated with and without JH295. Data are fold change in luminescence relative to the LPS control, are plotted as individual data points of three to six independent biological replicates using three to six unique cell donors, and analyzed using unpaired two-tailed *t* test. ns, not significant.

Next, we examined the effects of NEK2 inhibition on PEL by using the NEK2 inhibitor, JH295. JH295 is an irreversible and highly specific inhibitor of NEK2 that was previously shown to have no off-target effects on other mitotic kinases including polo-like kinase 1 (Plk1), cyclin-dependent kinase 1 (Cdk1), and Aurora B (16). We treated the panel of PEL cells with a range of JH295 concentrations (Fig. 2A) and determined IC_50_ values at 24, 48, and 72 hours posttreatment. Results demonstrated that all PEL cell lines were highly susceptible to JH295 treatment, with IC_50_ values in the low nanomolar range at all timepoints tested (Supplementary Table S1). While we noted similar IC_50_ values across the time course, PEL cell death peaked at 48 hours post-JH295 treatment (Supplementary Table S1). We also tested another NEK2 inhibitor, NCL-00017509, on the PEL cells. Unlike JH295, NCL-00017509 is a reversible NEK2 inhibitor (17). Results showed that the IC_50_ values for NCL-00017509 in PEL were substantially higher than for JH295 (Supplementary Table S2), indicating that the irreversible NEK2 inhibitor, JH295, was more effective in PEL.

We next quantified the effects that NEK2 inhibition had on PEL viability and propagation. We treated PEL cells with increasing amounts of JH295 or vehicle control (DMSO) and quantified cell death after 48 hours. The percentage of dead cells increased in a dose-dependent manner in all PEL cell lines tested (Fig. 2B). Using a Trypan blue assay, we also quantified the number of live cells present in each treatment group after 48 hours. In all PEL cell lines, live cell numbers significantly decreased in a dose-dependent manner following JH295 treatment (Fig. 2C). Taken together, these data suggest that NEK2 activity, which is blocked by JH295, is required for PEL survival and proliferation.

We next sought to determine the effects that NEK2 inhibition had on normal cells. We treated PBMCs isolated from healthy donors with the same range of JH295 concentrations tested on PEL and compared cell viability after 48 hours. Results showed that PEL cells were significantly more susceptible to JH295 treatment compared with PBMCs, and that NEK2 inhibition had little effect on PBMC viability (Fig. 2D and E). We then performed these same cell viability assays with B cells from healthy donors. Primary B cells were isolated from donor PBMCs and B-cell purity was confirmed via flow cytometry (Supplementary Fig. S1). We then treated the primary B cells with JH295 or vehicle control (DMSO) for 48 hours. No significant differences in viability were observed in primary B cells treated with JH295 compared with the DMSO control (Fig. 2F). However, considering that primary B cells do not normally divide and that NEK2 is a mitotic kinase, we wanted to verify these findings in the context of active B-cell proliferation. Thus, we treated primary B cells from healthy donors with LPS, which induced significant B-cell proliferation relative to mock-stimulated cells (PBS, Fig. 2G). We then treated these stimulated B cells with or without JH295 and, once again, we saw no differences in viability of the JH295-treated stimulated B cells relative to the untreated stimulated B cells 48 hours later (Fig. 2H), indicating that NEK2 inhibition does not affect the viability of resting or activated B cells. Altogether, our data suggest that JH295 treatment results in the death of PEL cells but not normal primary B cells, regardless of proliferation status.

### PEL Cells Undergo Caspase 3–Mediated Apoptosis Following NEK2 Inhibition

**FIGURE 3:**
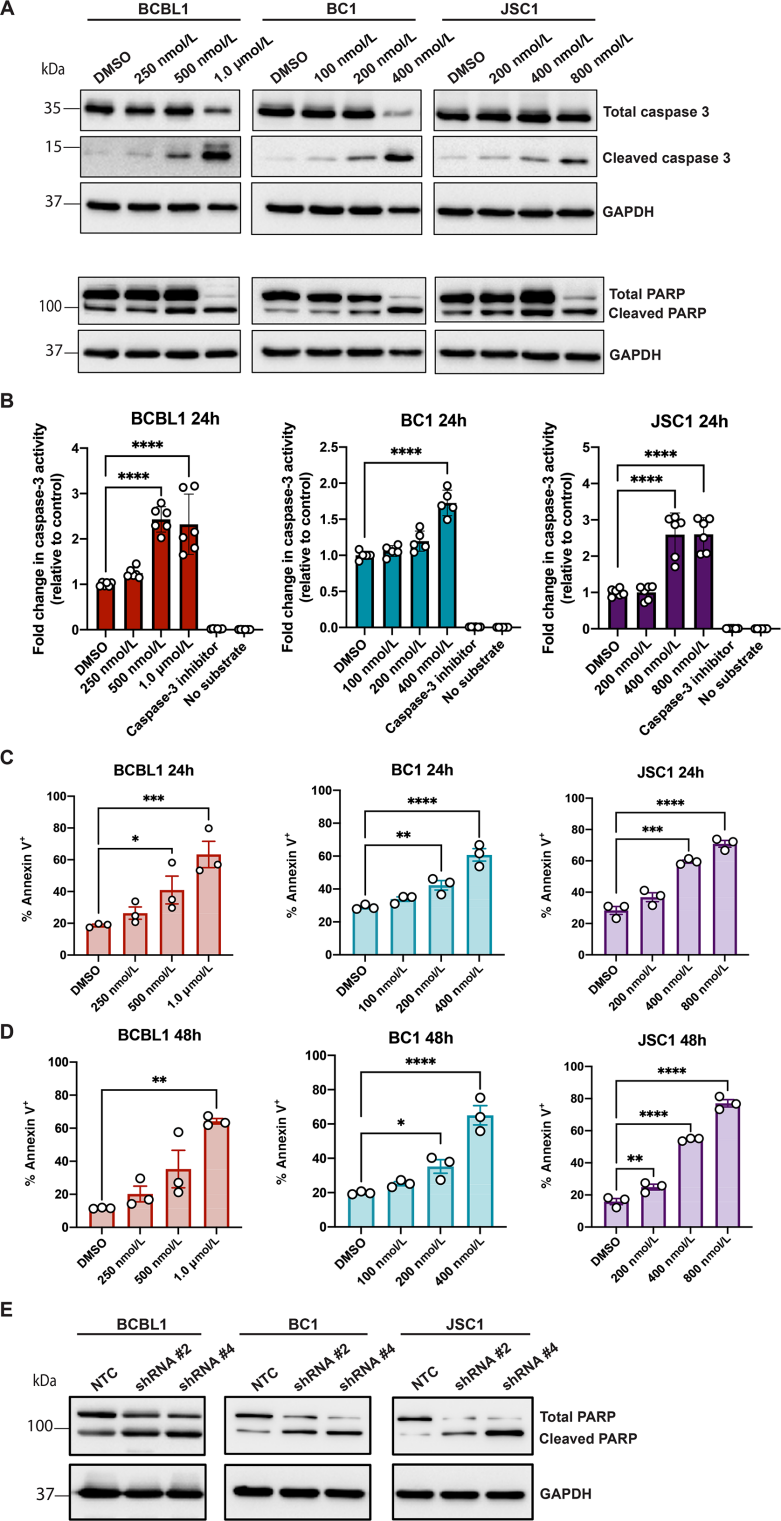
PEL cells undergo caspase 3–mediated apoptosis following NEK2 inhibition. **A,** Western blot analysis for caspase 3 and PARP in PEL cells treated with and without JH295. GAPDH was used as the loading control. Data are representative of three independent biological replicates. **B,** Caspase 3 enzymatic activity assay in PEL cells treated with and without JH295 relative to the DMSO control. Data are plotted as individual values from three independent biological replicates, are represented as mean ± SD, and analyzed using one-way ANOVA with Dunnett multiple comparisons. ****, *P* < 0.0001. **C** and **D,** Annexin V positivity in PEL cells after 24 hours (C) and 48 hours (D) of JH295 treatment, as measured by flow cytometry. Data are from three independent biological replicates, are represented as mean ± SEM, and analyzed using two-way ANOVA with Dunnett multiple comparisons. ****, *P* < 0.0001. BCBL1 24 hours ***, *P* = 0.0006; *, *P* = 0.02; BC1 24 hours **, *P* = 0.0045; JSC1 24 hours ***, *P* = 0.0002; BCBL1 48 hours **, *P* = 0.0015; BC1 48 hours *, *P* = 0.0118; JSC1 48 hours **, *P* = 0.0035. **E,** Western blot for PARP in PEL cells with intact NEK2 expression (NTC) or depleted NEK2 expression (shRNA). GAPDH was used as the loading control. Data are representative of three independent biological replicates. The BCBL1 loading control blot in E is the same as in Fig. 1C.

We next determined the mechanism of cell death occuring in the PEL cells after NEK2 inhibition. As PEL cells are positive for KSHV, we first tested whether NEK2 inhibition induced lytic reactivation of the virus, leading to PEL cell death. However, when we treated the PEL cells with or without JH295 for 48 hours and measured KSHV lytic gene expression via qPCR, we did not observe any appreciable increase in viral lytic gene transcripts following JH295 treatment (Supplementary Fig. S2A). These results were recapitulated in PEL cells with depleted NEK2 protein (Supplementary Fig. S2B and S2C). Expression of the KSHV immediate early protein, ORF45, was similar in PEL cells with both intact and depleted NEK2 expression (Supplementary Fig. S2B), corroborating the KSHV lytic gene qPCR data (Supplementary Fig. S2C). Together, these data suggest that JH295 treatment does not induce KSHV reactivation in PEL. To determine whether an intrinsic form of cell death was instead occuring in PEL following NEK2 inhibition, PEL cells were treated with DMSO or increasing concentrations of JH295 for 48 hours and protein was extracted from whole-cell lysates. Examination via Western blot revealed that expression of cleaved caspase 3, as well as cleaved PARP, increased in a dose-dependent manner in PEL after JH295 treatment (Fig. 3A). To validate these findings, we quantified the activity of the caspase 3 protease in the PEL cells after treatment with and without JH295 and found that caspase 3 activity was significantly elevated in all PEL cell lines treated with JH295 compared with the DMSO control (Fig. 3B). As cleavage of PARP and activation of caspase 3 are both indicative of apoptotic cell death, we quantified the number of apoptotic cells using annexin V staining of PEL cells following treatment with and without JH295. At both 24 and 48 hours posttreatment, the percentage of annexin V–positive cells was significantly elevated in a dose-dependent manner in all PEL cell lines (Fig. 3C and D). Furthermore, we also detected a similar induction of PARP cleavage in PEL cells with depleted NEK2 expression relative to control cells with intact NEK2 expression (Fig. 3E). Taken together, these data demonstrate that PEL cells undergo caspase 3–mediated apoptotic cell death in the absence of NEK2 signaling and further support the prosurvival role of NEK2 in PEL.

To further address the feasibility and efficacy of NEK2 inhibition in PEL, we wanted to compare our findings with JH295 to another inhibitor, T-1101 tosylate, currently in phase I trials for refractory solid tumors (18, 19). T-1101 tosylate disrupts the interaction of NEK2 with one of its target proteins, Highly Expressed in Cancer 1 (Hec1), and therefore partially inhibits the NEK2 signaling pathway and induces apoptosis in cancer cells (16, 18). We found that treatment with T-1101 tosylate reduced PEL viability over time compared with vehicle treatment (DMSO, Supplementary Fig. S3A–S3C). In addition, while JH295 acted more rapidly in PEL than T-1101 tosylate, the IC_50_ values for T-1101 tosylate were in the low nanomolar range and similar to those of JH295 (Supplementary Tables S1 and S3). A comparison of the JH295 and T-1101 tosylate treatment with respect to IC_50_ and viability of PEL is shown in Supplementary Fig. S3D and S3E.

### JH295 Treatment Results in G_1_ Cell-Cycle Arrest in PEL

**FIGURE 4:**
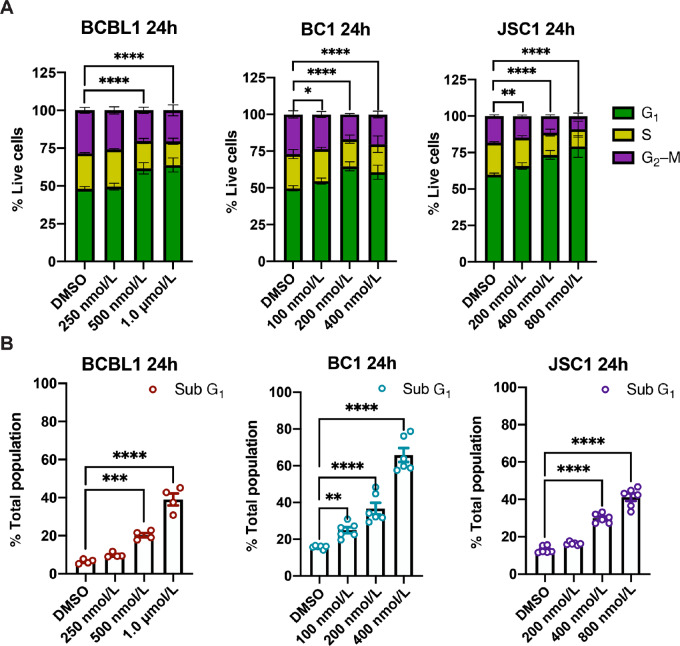
JH295 treatment results in G_1_ stage cell-cycle arrest in PEL. **A,** Cell-cycle analysis via PI staining and flow cytometry of PEL cells treated with or without JH295. Data represent mean ± SD of three independent biological replicates and were analyzed for the G_1_ cell population using two-way ANOVA with Dunnett multiple comparisons. ****, *P* < 0.0001. BC1 24 hours *, *P* = 0.0172; JSC1 24 hours **, *P* = 0.0039. **B,** Quantification of the sub-G_1_ population in PEL cells treated with or without JH295. Data are from the same experiments as A, are plotted as individual data points representing mean ± SEM, and analyzed using two-way ANOVA with Dunnett multiple comparisons. Data are from three independent biological replicates, each performed in duplicate except BCBL1, which was performed in duplicate once and in single replicates twice. ****, *P* < 0.0001. BCBL1 ***, *P* = 0.0003; BC1 24 hours **, *P* = 0.0061.

Considering NEK2 is a mitotic kinase, we next examined cell-cycle progression in PEL following NEK2 inhibition. PEL cells were treated with JH295 or vehicle control (DMSO) for 24 hours and then cell-cycle analysis was performed using PI staining and flow cytometry. We found that PEL cells undergo slight but significant G_1_ phase arrest following JH295 treatment (Fig. 4A). In addition, we found that the proportion of cells in sub-G_1_ phase increased in a dose-dependent manner following JH295 treatment in all PEL cell lines (Fig. 4B).

### NEK2 Inhibition Modulates Cellular Protein Expression

**FIGURE 5:**
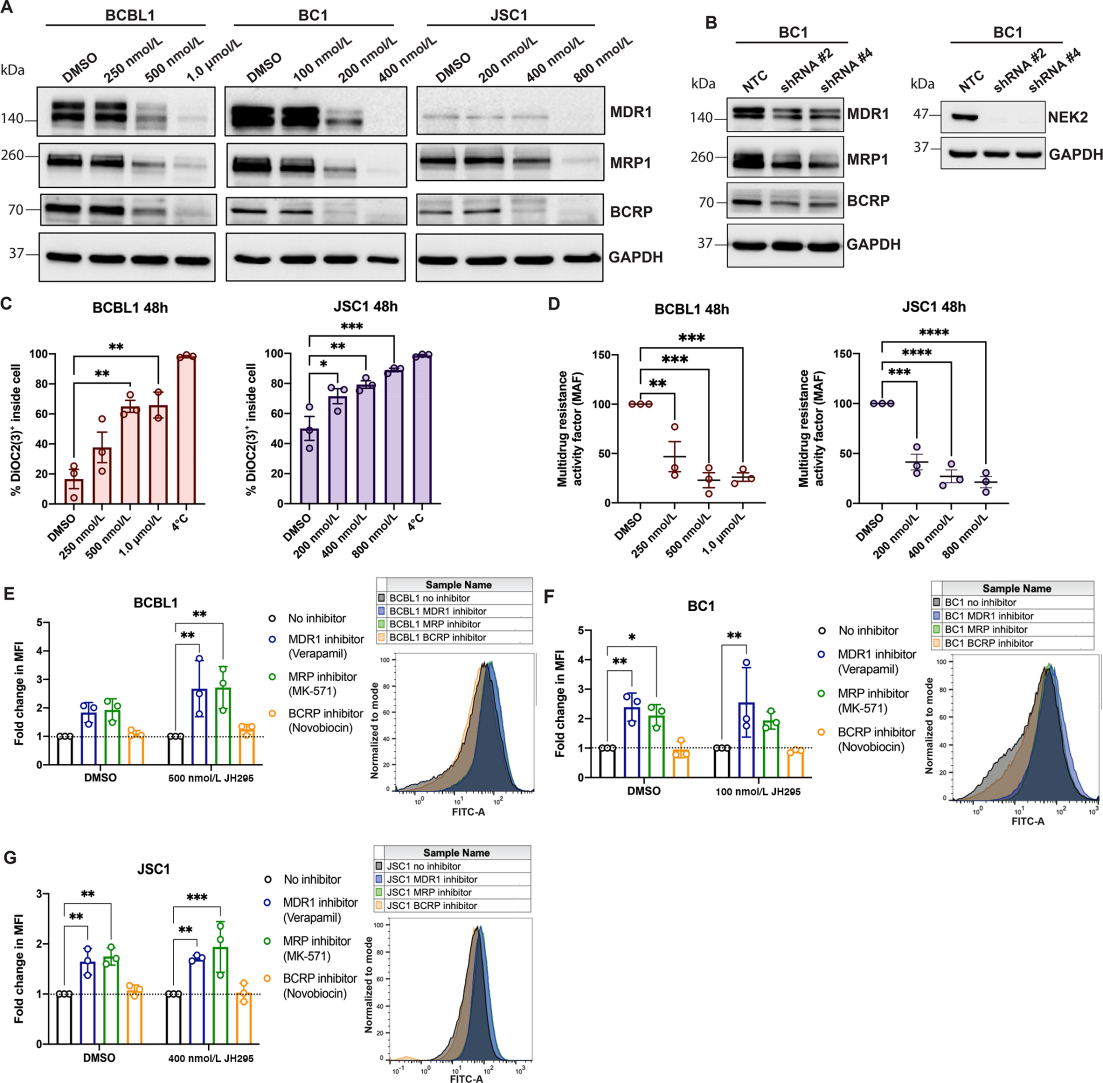
NEK2 inhibition in PEL decreases expression and activity of the ABC transporter proteins, MDR1 and MRP. **A,** Western blot analysis for MDR1, MRP1, and BCRP in PEL cells treated with or without JH295. GAPDH was used as the loading control. Data are representative of three independent biological replicates. **B,** Western blot analysis for MDR1, MRP1, and BCRP in BC1 cells with intact NEK2 expression (NTC) or depleted NEK2 expression (shRNA). The accompanying Western blot analysis demonstrates depleted NEK2 expression in the BC1 cells used. GAPDH was used as the loading control. All data are representative of three independent biological replicates. **C,** Multidrug resistance assay measuring the activity of ABC transporter proteins in PEL cells treated with or without JH295. Data represent the percentage of fluorescent dye retained inside the cells after the efflux period. The 4°C sample represents a positive control (no activity; near 100% dye retention within the cells). Data are plotted as individual data points of three independent biological replicates representing mean ± SEM and analyzed using one-way ANOVA with Dunnett multiple comparisons. ***, *P* = 0.0003; **, *P* < 0.004; *, *P* = 0.02. **D,** Data are from the same experiments as C but used to calculate the multidrug resistance activity factor (MAF) for each treatment condition. Data are plotted as individual data points of three independent biological replicates representing mean ± SEM and analyzed using one-way ANOVA with Dunnett multiple comparisons. BCBL1 48 hours ***, *P* < 0.001; **, *P* = 0.0069; JSC1 48 hours ****, *P* < 0.0001; ***, *P* = 0.0003. **E**–**G,** eFLUXX-ID Green assay measuring the contribution of each individual ABC transporter protein to efflux activity in PEL cells treated with or without JH295 for 48 hours. Data are fold change in mean fluorescence intensity (MFI) relative to the no inhibitor control. Data are plotted as individual data points of three independent biological replicates representing mean ± SD and analyzed using two-way ANOVA with Dunnett multiple comparisons. BCBL1 **, *P* < 0.002; BC1 **, *P* < 0.009, *, *P* = 0.0352; JSC1 ***, *P* = 0.0003, **, *P* < 0.008. Corresponding histogram plots of FITC (green fluorescent dye) intensity within the PEL cells are gated on PI-negative single cells, normalized to mode, and are representative of three independent biological replicates.

To further understand the impact of NEK2 inhibition on PEL protein expression, we performed immunoblotting for proteins downsteam of the NEK2 signaling pathway. β-Catenin is one such protein involved in cellular proliferation and drug resistance, and is activated by NEK2 via direct phosphorylation (20). We found that expression of total, as well as phosphorylated, β-catenin decreased in the PEL cells after JH295 treatment (Supplementary Fig. S4A). We also observed decreased phosphorylation of β-catenin in a majority of PEL cell lines with depleted NEK2 protein expression (Supplementary Fig. S4B), together suggesting that NEK2 inhibition results in decreased β-catenin activation. Expression of the prosurvival protein, B-cell lymphoma extra large (Bcl-xL), was also found to be decreased in the PEL cells upon JH295 treatment as well as in a majority of the NEK2-depleted PEL cell lines (Supplementary Fig. S4A and S4B), suggesting that Bcl-xL acts to help prevent cell death during intact NEK2 signaling. Indeed, treatment of PEL with the Bcl-xL–specific inhibitor, A-1155463, resulted in cell death (Supplementary Fig. S4C). Interestingly, in addition to β-catenin and Bcl-xL, we found that expression of three major ATP binding cassette (ABC) transporter proteins, MDR1, MRP1, and breast cancer resistance protein (BCRP), strikingly decreased in a dose-dependent manner following JH295 treatment in the PEL cells (Fig. 5A). ABC transporter proteins are membrane-bound proteins that facilitate movement of various substrates out of cells via ATP hydrolysis (21). Many of these substrates are toxic to the cell, and therefore ABC transporter proteins play a key role in the maintenance and survival of normal cells throughout the body (22). However, malignant cells can take advantage of this mechanism by upregulating ABC transporter protein expression to expel anticancer drugs from the cells, leading to tumor persistence and drug resistance (23). Our observed reduction in ABC transporter protein expression in PEL following JH295 treatment was recapitulated in the NEK2 knockdown cells (Fig. 5B). Altogether, these data demonstrate that inhibition and/or depletion of NEK2 in PEL results in decreased expression of proteins involved in cell survival and drug resistance.

### The ABC Transporter Proteins MDR1 and MRP Mediate Drug Efflux in PEL

To determine whether the observed decrease in ABC transporter protein expression in PEL following JH295 treatment carried functional significance, we performed a Multidrug Resistance Direct Dye Efflux Assay on PEL cells treated with and without JH295. In this assay, efflux of a fluorescent dye, DiOC_2_(3), from cells via ABC transporter protein activity is quantified by flow cytometry. As ABC transporter proteins are active at 37°C, a sample loaded with dye and maintained at 4°C throughout the duration of the assay serves as a positive control (i.e., ∼100% of the loaded dye maintained within the cells). Results of this assay showed a dose-dependent decrease in ABC transporter protein activity [i.e., a dose-dependent increase in DiOC_2_(3) positivity inside the cell] following JH295 treatment in PEL (Fig. 5C), indicating that NEK2 inhibition not only decreases expression of these drug transporter proteins but also inhibits their function. Using these efflux data, we then calculated the multidrug resistance activity factor (MAF) of PEL following treatment with and without JH295 and found that the MAFs significantly decrease with NEK2 inhibition (Fig. 5D). Taken together, these data demonstrate that NEK2 inhibition reduces expression and efflux activity of ABC transporter proteins in PEL and lowers the drug resistance propensity of PEL.

We next wanted to measure the contribution of each ABC transporter protein (MDR1, MRP, and BCRP) to efflux activity in PEL. To measure efflux through each individual transporter protein, we used the eFLUXX-ID Green Multidrug Resistance Assay. This assay utilizes a proprietary green dye that becomes fluorescent upon cleavage by intracellular esterases and is then removed from cells by ABC transporter proteins. PEL cells treated with DMSO or JH295 were incubated with and without individual inhibitors specific for each transporter protein prior to addition of the green dye. Verapamil was used to inhibit MDR1 activity, MK-571 was used to inhibit MRP activity, and novobiocin was used to inhibit BCRP activity. After allowing time for the dye to efflux out of the cells, flow cytometry was used to measure the activity of MDR1, MRP, and BCRP by quantifying the amount of dye retained within the cells post-efflux. We found that MDR1 and MRP are the ABC transporter proteins most active in removing substrates from PEL cells, while BCRP has little activity (Fig. 5E–G). We observed the same phenotypes in PEL cells treated with JH295 as well as DMSO. Overall, our data suggest that MDR1 and MRP are the main mediators of drug efflux in PEL.

### NEK2 Inhibition Reduces PEL Burden and Prolongs Survival *In Vivo*

**FIGURE 6:**
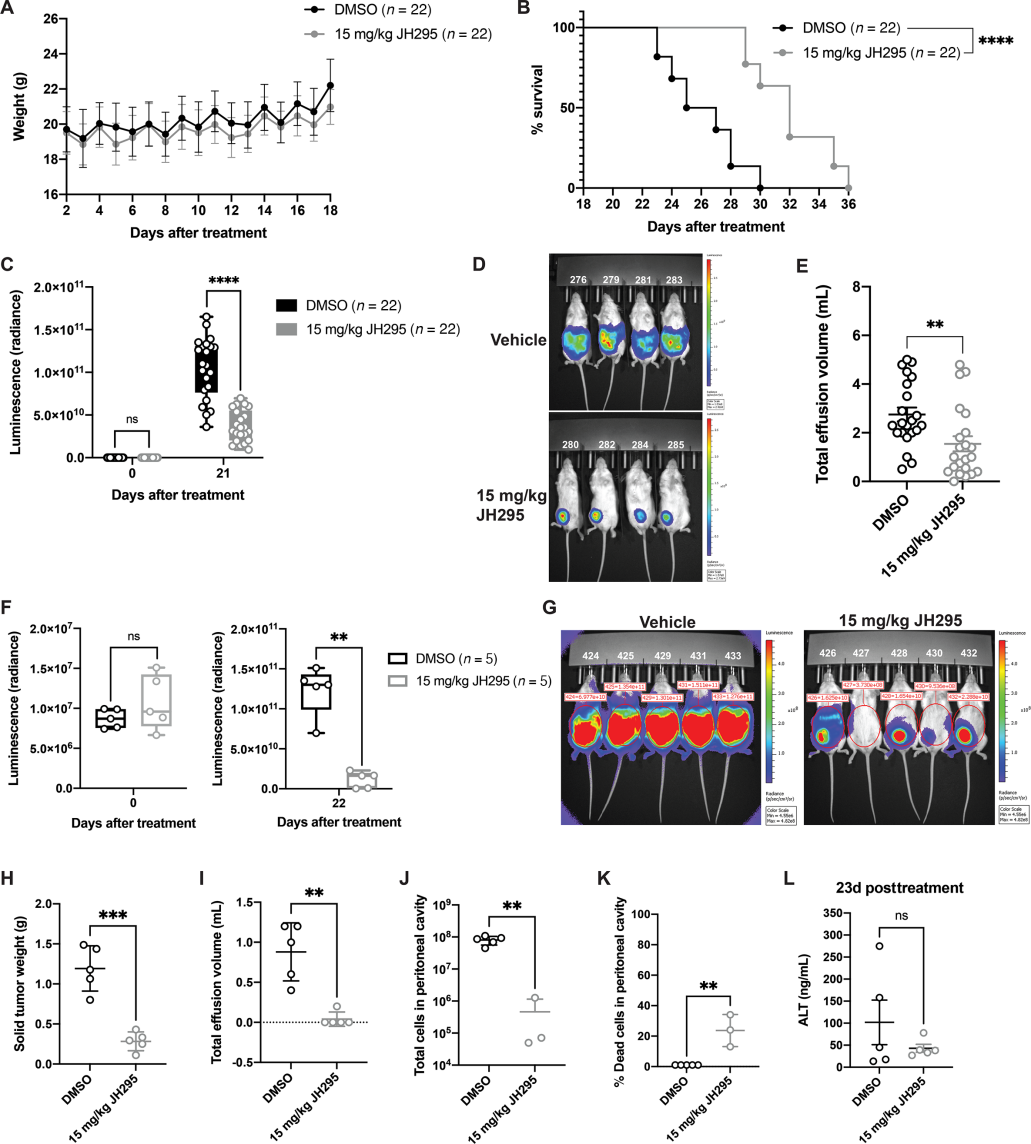
NEK2 inhibition decreases tumor burden and prolongs survival in PEL xenograft mice. **A,** Weights of PEL-bearing mice over time treated with vehicle control (DMSO, black line) or 15 mg/kg JH295 (gray line). Data represent mean ± SD. **B,** Survival curves of PEL-bearing mice treated with DMSO (black line) or 15 mg/kg JH295 (gray line). Data were analyzed using log-rank (Mantel–Cox) test. ****P < 0.0001. **C,** Quantification of PEL burden (as measured by luminescence) in mice treated with DMSO or 15 mg/kg JH295. Data were analyzed using multiple unpaired *t* tests. ns, not significant, ****, *P* < 0.0001. **D,** Representative IVIS imaging of PEL burden in mice treated with vehicle (DMSO) or 15 mg/kg JH295 at 21 days posttreatment. **E,** Quantification of total effusion volume isolated from the peritoneal cavity of PEL-bearing mice at endpoint treated with either DMSO or 15 mg/kg JH295. Data were analyzed using unpaired two-tailed *t* test. **, *P* = 0.0066. All data (unless otherwise stated) are pooled from three independent biological replicates. **F,** Quantification of PEL burden (as measured by luminescence) in mice treated with DMSO or 15 mg/kg JH295. Data were analyzed using paired two-tailed *t* test. ns, not significant; **, *P* = 0.0025. **G,** IVIS imaging of PEL burden in mice treated with vehicle (DMSO) or 15 mg/kg JH295 at 22 days posttreatment. Red circles indicate the region of interest, and tumor burden (as measured by luminescence) is indicated above each mouse (same data in F). **H,** Weight of solid tumors isolated from PEL-bearing mice treated with either DMSO or 15 mg/kg JH295 at 23 days posttreatment. Data were analyzed using unpaired two-tailed t-test. ***P = 0.0002. **I,** Quantification of total effusion volume isolated from the peritoneal cavity of PEL-bearing mice at 23 days posttreatment. Data were analyzed using unpaired two-tailed *t* test. **, *P* = 0.001. **J** and **K,** Total number of cells (**J**) and % dead cells (K) isolated 23 days posttreatment from the peritoneal cavities of PEL-bearing mice treated with either DMSO or 15 mg/kg JH295. For 4/5 JH295-treated mice, no effusion was present, so cells were collected via peritoneal lavage using 1 mL PBS. Two samples in the JH295 treatment group were contaminated with red blood cells and therefore omitted. Data were analyzed using unpaired two-tailed *t* test. **, *P* < 0.0025. **L,** Quantification of ALT present in the sera of PEL-bearing mice at 23 days posttreatment. Data were analyzed using unpaired two-tailed *t* test. ns, not significant.

After characterizing the effects of JH295 treatment on PEL *in vitro*, we evaluated the efficacy of NEK2 inhibition via JH295 treatment on PEL *in vivo* using a PEL xenograft mouse model, in which PEL burden over time can be monitored in mice by live imaging (6). Six- to 8-week-old NOD/SCID/gamma (NSG) mice were injected with 70,000–100,000 BCBL1-luciferase cells which can be imaged *in situ* via luciferin injection. Three days post PEL cell injection, mice were randomized according to PEL burden and treated with 15 mg/kg JH295 or vehicle control (DMSO) intraperitoneally 3×/week until humane endpoints were reached. JH295 treatment did not affect the weights of the mice over time (Fig. 6A); however, mice treated with JH295 survived significantly longer than the vehicle-treated mice (Fig. 6B), suggesting that NEK2 inhibition prolongs host survival in PEL. Systemic PEL burden, as measured by luminescence, was significantly reduced in the JH295-treated animals compared with the control animals at 21 days posttreatment (Fig. 6C and D). PEL effusions (ascites) collected from the peritoneal cavity at endpoint were also significantly reduced in the JH295-treated animals compared with the vehicle animals (Fig. 6E). Overall, these data demonstrate that NEK2 inhibition via JH295 treatment significantly prolongs survival and reduces tumor burden in PEL-bearing mice.

To more directly assess the effects of JH295 on PEL tumor burden, we performed an additional experiment where PEL-bearing mice were treated with DMSO or 15 mg/kg JH295 as described above and then all animals were humanely euthanized at the same timepoint (23 days posttreatment). We again observed a significant decrease in overall tumor burden in the JH295-treated mice compared with the control mice (Fig. 6F and G, 22 days posttreatment). Solid tumors were collected from the mice and weighed, and results showed a significant decrease in solid tumor weight when JH295 had been administered compared with DMSO (Fig. 6H). Ascites were also significantly reduced in the JH295-treated mice compared with the control mice (Fig. 6I) with only 1/5 of JH295-treated mice developing ascites compared with 5/5 vehicle mice. In mice that did not develop ascites, cells were collected from the peritoneal cavity via PBS lavage. We then performed Trypan blue counts on the cells isolated from the peritoneal cavities of the animals and found that the total number of cells, as well as the percentage of viable cells, were significantly higher in the control mice compared with the mice treated with JH295 (Fig. 6J and K). Finally, to assess potential toxicity of JH295 *in vivo*, we quantified the levels of alanine transaminase (ALT) in the sera of the mice 23 days posttreatment as a proxy for liver function. No significant differences were observed in ALT levels between the control and JH295 groups (Fig. 6L), suggesting that JH295 is not harmful to the liver. Overall, these data demonstrate the potency of JH295 in reducing PEL tumor burden *in vivo* with no apparent liver toxicity.

### Inhibition of NEK2 Sensitizes Lymphomas to Chemotherapy and has a Synergistic Effect on Lymphoma Cell Death

**FIGURE 7:**
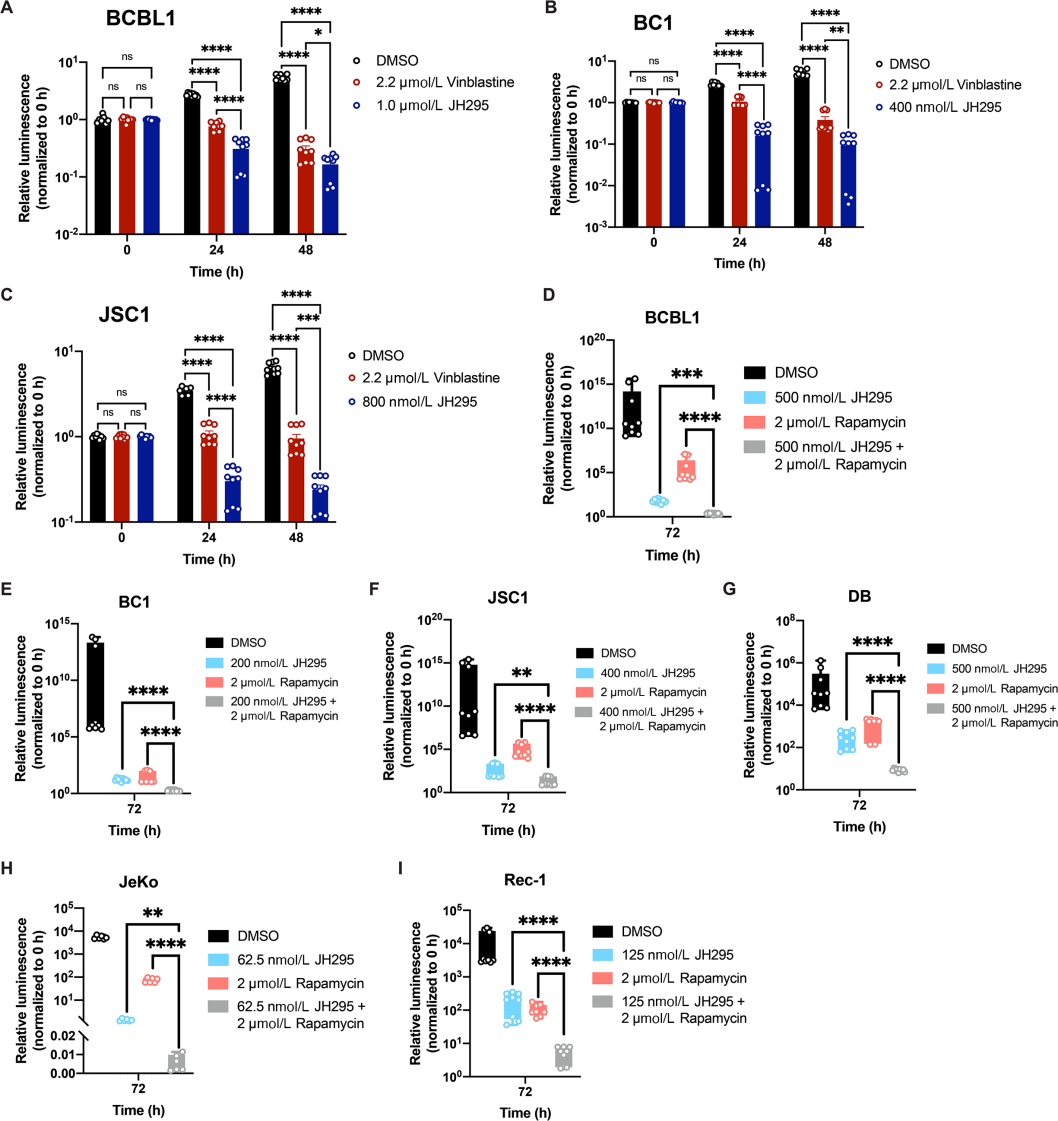
JH295 treatment sensitizes viral and non-viral lymphomas to chemotherapy and has a synergistic effect on lymphoma cell death. **A–C,** Viability of PEL cells over time treated with vehicle control (DMSO, black bars), 2.2 µmol/L vinblastine (red bars), or ≤ 1.0 µmol/L JH295 (navy bars). Data are plotted as individual values of three independent biological replicates performed in triplicate and normalized to the 0-hour luminescence values for each treatment group. Data represent mean ± SEM and were analyzed using two-way ANOVA with Tukey multiple comparisons. ns, not significant; ****, *P* < 0.0001; ***, *P* < 0.0006; **, *P* = 0.0092; *, *P* < 0.04. **D–I,** Viability of lymphoma cells following 72 hours of treatment with DMSO (black boxes), rapamycin (salmon boxes), JH295 (aquamarine boxes), or rapamycin + JH295 (gray boxes). Data are plotted as individual values of three independent biological replicates performed in triplicate and normalized to the corresponding 0-hour luminescence values. Data were analyzed using one-way ANOVA with Sidak multiple comparisons. ****, *P* < 0.0001; ***, *P* = 0.0002; **, *P* < 0.007. **D–F,** virus-associated PEL cell lines. **G,** Nonviral DLBCL cell line. **H–I,** Nonviral mantle cell lymphoma cell lines.

Because our data suggested that targeting NEK2 could be a therapeutic approach for PEL, we then compared the antitumor efficacy of JH295 treatment to that of vinblastine, a drug similar to vincristine, used clinically in PEL chemotherapeutic regimens (24). We treated PEL cells with either high-dose vinblastine (2.2 µmol/L) or lower concentrations of JH295 (0.4–1.0 µmol/L) and measured cell viability after 24 and 48 hours of drug treatment. For all PEL cell lines, JH295 treatment resulted in greater PEL cell death than vinblastine treatment at both time points tested (Fig. 7A–C), indicating that JH295 is more potent than vinblastine against PEL, even at lower concentrations.

Finally, considering the resistant nature of PEL to chemotherapy and our data demonstrating decreased expression and activity of ABC transporter proteins following JH295 treatment, we sought to determine whether JH295 administration could sensitize PEL to the mTOR inhibitor, rapamycin, an FDA-approved drug we previously reported for PEL (25). We treated PEL cells with DMSO, JH295 (0.2–0.5 µmol/L), rapamycin (2.0 µmol/L), or a combination of JH295 + rapamycin. We found that JH295 enhanced the efficacy of rapamycin, and combination therapy resulted in PEL cell death that was significantly greater than either drug treatment alone (Fig. 7D–F). To determine whether this synergistic effect of JH295 and rapamycin was specific to PEL or extended to nonviral lymphomas, we performed the same experiment using a KSHV- and Epstein–Barr virus (EBV)-negative diffuse large B-cell lymphoma (DLBCL) cell line (DB) and two mantle cell lymphoma cell lines (JeKo and Rec-1) that also exhibited upregulated NEK2 expression (6). Similar to our results in PEL, we observed a significant increase in lymphoma cell death when JH295 was combined with rapamycin compared with either monotherapy (Fig. 7G–I). Taken together, these data indicate that JH295 treatment can sensitize both viral and nonviral lymphomas to chemotherapeutics currently in clinical use, and that NEK2 inhibition represents a potent anti-PEL strategy as both a monotherapy and as part of a synergistic combination therapy.

## Discussion

To our knowledge, this is the first study describing the role of NEK2 in the growth and chemoresistance of viral lymphomas. Here, we show that genetic (shRNA) and pharmacologic (drug inhibitor) targeting of NEK2 results in apoptotic PEL cell death. In addition, in the absence of NEK2 signaling, PEL cells undergo G_1_ cell-cycle arrest and downregulate proteins involved in cell survival and drug resistance. Importantly, inhibition of NEK2 does not affect the survival of nonmalignant cells. We also demonstrate that treatment of PEL-bearing mice with the NEK2 inhibitor, JH295, significantly reduces tumor burden and prolongs survival compared with control mice, with no harmful effects on liver health. Finally, we show that JH295 can sensitize both viral and nonviral lymphomas to chemotherapy with synergistic effects on cell death. Together, these data suggest that NEK2 represents a potent target for the treatment of PEL, and that JH295 administration could potentially help overcome the problem of drug resistance in lymphoma therapy.

A prominent concern in cancer therapy when targeting proteins that are involved in cell-cycle progression is the off-target effects this might have on normal and, especially, proliferating cells. We show that treatment of PBMCs and primary B cells with JH295 does not significantly affect viability and that this is the case even for proliferating primary B cells. These data suggest that, even though NEK2 is a mitotic kinase, inhibition of NEK2 in healthy proliferating cells does not have negative effects on cell viability. Therefore, NEK2 function is critical for the survival of PEL, but seemingly dispensible for the survival of normal cells. This could be due to compensatory function by another kinase in healthy cells. Alternatively, it is also possible that PEL cells, are so reliant on NEK2 for survival that they are unable to compensate for the loss of NEK2 function and instead undergo apoptosis. Our *in vivo* data also suggest NEK2 inhibition is not detrimental to overall host health. Altogether, our data suggest that PEL exibits a unique dependency on NEK2 function for survival compared with normal cells, and that JH295 may therein posess an inherent selectivity for PEL.

The mechanism of cell death in PEL following NEK2 inhibition is likely multifactorial, including intrinsic apoptosis driven by caspase 3 activation, loss of Bcl-xL expression, and cell-cycle arrest. The downregulation of ABC transporter proteins post-NEK2 inhibition could also be contributing to PEL cell death, as these proteins help to maintain cellular homeostasis (26). Although all tested PEL cell lines were susceptible to NEK2 inhibition, the IC_50_ value for JH295 was highest in the BCBL1 cell line. This could be due to proliferation rate, as BCBL1 cells grow more quickly than BC1 and JSC1 cells. We have also observed that BCBL1 cells tend to be more aggressive and are more resistant to chemotherapy compared with other PEL (27).

We observed not only was ABC transporter protein expression decreased in PEL following JH295 treatment, but functional activity of these proteins was decreased as well, suggesting a clinical significance of our findings. Indeed, drug resistance in cancer is often mediated by ABC transporter proteins (including MDR1, MRP, and BCRP) actively pumping chemotherapeutic agents out of tumor cells (28–32). Over the last three decades, multiple attempts at targeting this drug resistance mechanism in patients with cancer have been made (33–39) but, unfortunately, many of these therapies have failed in clinical trials, in part due to negative side effects likely caused by toxicity of the compounds in use (40, 41). Therefore, it would be of great interest to identify a drug that had limited off-target effects in healthy cells while actively blocking the efflux activity of ABC transporter proteins. Herein, we have identified JH295 as a compound potentially capable of fulfilling these roles. It is possible that JH295 treatment causes a build-up of JH295 itself within PEL cells due to inhibition of ABC transporter protein activity, and that this accumulation of JH295 causes PEL cells to undergo apoptosis. Another potential mechanism is that inhibition of ABC transporter proteins via JH295 treatment causes toxic by-products to accumulate within the PEL cells, resulting in cell death. To help test this hypothesis, we quantified free cholesterol levels within the PEL cells after mock and JH295 treatment, as free cholesterol is known to be toxic to mammalian cells and is removed from cells via ABC transporter activity (42–45). However, we found no significant differences in free cholesterol levels in PEL cells treated with or without JH295. This could be in part due to cancer cells containing high baseline levels of free cholesterol (46–49), and therefore PEL cells could already be saturated with free cholesterol prior to NEK2 inhibition.

To our knowledge, this is the first report to quantify the activity of each major ABC transporter protein in PEL. We have shown that MDR1 and MRP are most active in PEL, while BCRP has little activity. Interestingly, we observed roughly the same level of efflux disruption in PEL cells when either MDR1 or MRP was inhibited, suggesting that, although both MDR1 and MRP have activity in PEL, single inhibition of either protein is sufficient to significantly disrupt efflux activity, perhaps due to nonredundant functions of the two transporter proteins. A previous study demonstrated that the inhibitor, MK-571, was highly specific for MRP and had no effect on MDR1 activity in acute myeloid leukemia cells (50), indicating that simultaneous dual inhibition of MDR1 and MRP by MK-571 is unlikely. Although it has previously been reported that verapamil can downregulate MRP expression in gallbladder cancer cells (51), in our assays the cells are exposed to verapamil for only a short period of time and it is therefore unlikely that protein expression is altered by verapamil treatment to any appreciable extent. We observed this same level of efflux disruption in PEL with either MDR1 or MRP inhibition in both the presence and absence of JH295 across all PEL cell lines tested, indicating that this is a property inherent to PEL and not simply a result of NEK2 inhibition.

Previous work in our laboratory examined the impact of the mTOR inhibitor, rapamycin, on PEL (25). Herein, we found that JH295 was more effective than rapamycin at reducing PEL viability at much lower drug concentrations, and that dual treatment of PEL with JH295 and rapamycin resulted in significantly more PEL death than either therapy alone. We also demonstrate that these findings extend to nonviral lymphomas, as similar phenotypes were observed in KSHV- and EBV-negative DLBCL and mantle cell lymphoma cell lines. Together, these data suggest that NEK2 inhibition via JH295 administration sensitizes PEL and other NHLs to rapamycin therapy and that these drugs act synergistically to increase lymphoma death. Therefore, JH295 could potentially be used as part of a synergistic chemotherapeutic regimen against multiple lymphomas.

In conclusion, we have identified NEK2 as an additional therapeutic target in PEL, and demonstrated that inhibition of NEK2 with the drug, JH295, exhibited potent and selective antilymphoma effects. Considering the refractory nature of PEL to current cancer drugs and the decreased drug resistance signatures we observed in PEL following JH295 treatment, NEK2 inhibition may represent a promising approach for clinical management of this malignancy.

## Supplementary Material

Supplementary Figure 1 Figure S1. Purity of primary B cells isolated from bulk PBMCs. (A) Flow cytometry analysis of primary B cell purity before (bulk PBMCs) and after (purified B cells) B cell purification from donor PBMCs using the STEMCELL EasySep system. Analyzed populations were gated on single cells and B cells were denoted as CD20-positive CD3-negative, while T cells were denoted as CD3-positive CD20-negative.

Supplementary Figure 2 Figure S2. NEK2 inhibition does not induce reactivation of KSHV. (A) Fold change in gene expression of KSHV lytic genes in PEL cells treated with or without JH295 for 48h. Data were normalized to actin and plotted as fold change relative to the DMSO control. For the positive control, BCBL1 cells were treated with DMSO and chemically reactivated with valproic acid for 48h. Data represent mean ± SEM of two biological replicates, each performed in triplicate, with the exception of the positive control, which is one biological replicate performed in triplicate. (B) Western blot for NEK2 and KSHV ORF45 in PEL cells with intact NEK2 expression (NTC) or depleted NEK2 expression (NEK2 siRNA). GAPDH was used as the loading control. Data are representative of two independent biological replicates. (C) Fold change in gene expression of KSHV lytic genes in PEL cells with intact NEK2 expression (NTC) or depleted NEK2 expression (NEK2 siRNA). Data were normalized to actin and plotted as fold change relative to the NTC. For the positive control, NTC-treated BCBL1 cells were chemically reactivated with valproic acid for 48h. Data represent mean ± SEM of two biological replicates, each performed in triplicate, with the exception of the positive control, which is one biological replicate performed in triplicate.

Supplementary Figure 3 Figure S3. Effects of the Hec1-NEK2 inhibitor, T-1101 tosylate, on PEL viability. (A-C) Viability of PEL cells treated with vehicle control (DMSO, black line) or various concentrations of T-1101 tosylate over time. Data were normalized to the 0h luminescence values for each treatment and represent mean ± SEM. Curves were fitted using non-linear regression and represent three independent biological replicates, each performed in triplicate. (D) IC50 curves of PEL cells treated with various concentrations of T-1101 tosylate or JH295 for 72h and normalized to the DMSO control values. Individual data points are plotted for two-three independent biological replicates, each performed in triplicate. Curves were fitted using non-linear regression and are connected by means. See also Table S3. (E) Viability of PEL cells treated with DMSO, 500 nM T-1101 tosylate, or 500 nM JH295. Data are plotted as individual data points of three independent biological replicates performed in triplicate, normalized to the DMSO control values, and represent mean ± SEM. Data were analyzed using two-way ANOVA with Šídák’s multiple comparisons. ****p < 0.0001. The DMSO data in both graphs in (E) are the same.

Supplementary Figure 4 Figure S4. NEK2 inhibition in PEL results in decreased expression of the transcription factor, beta-catenin, and the pro-survival protein, Bcl-xL. (A) Western blot for total beta-catenin, phosphorylated beta-catenin (Ser33/Ser37/Thr41, ~92 kDa band), and Bcl-xL in PEL cells treated with or without JH295 for 48h. GAPDH was used as the loading control. Data are representative of three independent biological replicates. (B) Western blot for phosphorylated beta-catenin (Ser33/Ser37/Thr41) and Bcl-xL in PEL cells with intact NEK2 expression (NTC) or depleted NEK2 expression (shRNA). GAPDH was used as the loading control. Data are representative of two-three independent biological replicates. (C) Viability of PEL cells treated with vehicle control (DMSO) or the Bcl-xL inhibitor, A-1155463, for 48h. Data are plotted as individual values from three-four independent biological replicates and normalized to the DMSO control values for each treatment. Data represent mean ± SD and were analyzed using two-way ANOVA with Dunnett’s multiple comparisons. ****p < 0.0001.

Supplementary Table 1 Table S1. JH295 IC50 values in PEL cell lines

Supplementary Table 2 Table S2. NCL-00017509 IC50 values in PEL cell lines

Supplementary Table 3 Table S3. T-1101 tosylate IC50 values in PEL cell lines
