# Supplementary material for: Inhibition of NEK2 Promotes Chemosensitivity and Reduces KSHV-positive Primary Effusion Lymphoma Burden
Source: Cancer Res Commun. 2024 Apr 9;4(4):1024–40. doi: 10.1158/2767-9764.CRC-23-0430 (PMC11003453; doi:10.1158/2767-9764.CRC-23-0430)
Supplement: Supplementary Figure 1 — Figure S1. Purity of primary B cells isolated from bulk PBMCs. (A) Flow cytometry analysis of primary B cell purity before (bulk PBMCs) and after (purified B cells) B cell purification from donor PBMCs using the STEMCELL EasySep system. Analyzed populations were gated on single cells and B cells were denoted as CD20-positive CD3-negative, while T cells were denoted as CD3-positive CD20-negative. [file crc-23-0430-s01.docx]

**
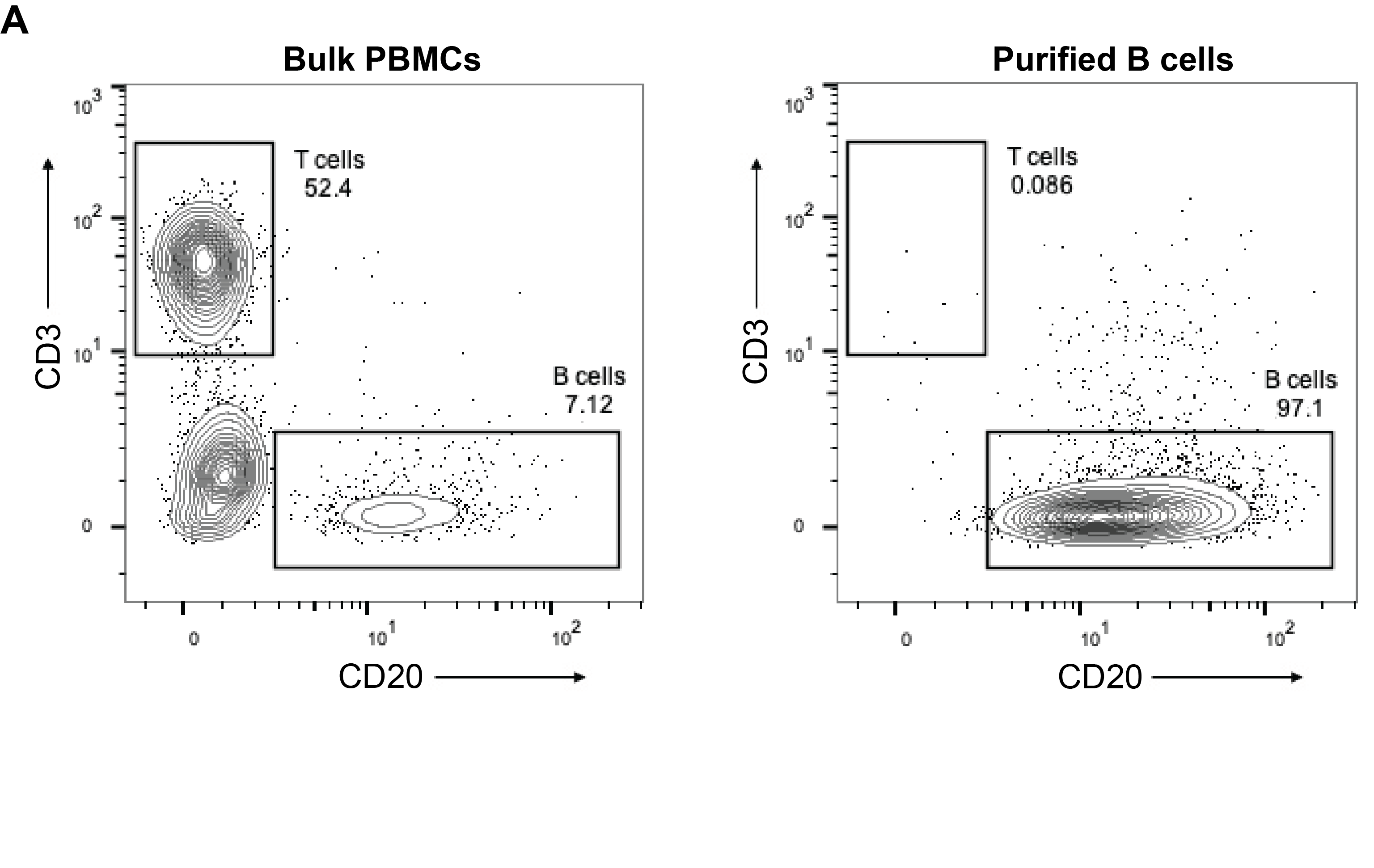
Figure S1. Purity of primary B cells isolated from bulk PBMCs.** (A) Flow cytometry analysis of primary B cell purity before (bulk PBMCs) and after (purified B cells) B cell purification from donor PBMCs using the STEMCELL EasySep system. Analyzed populations were gated on single cells and B cells were denoted as CD20-positive CD3-negative, while T cells were denoted as CD3-positive CD20-negative.
