# Supplementary material for: Inhibition of NEK2 Promotes Chemosensitivity and Reduces KSHV-positive Primary Effusion Lymphoma Burden
Source: Cancer Res Commun. 2024 Apr 9;4(4):1024–40. doi: 10.1158/2767-9764.CRC-23-0430 (PMC11003453; doi:10.1158/2767-9764.CRC-23-0430)
Supplement: Supplementary Figure 2 — Figure S2. NEK2 inhibition does not induce reactivation of KSHV. (A) Fold change in gene expression of KSHV lytic genes in PEL cells treated with or without JH295 for 48h. Data were normalized to actin and plotted as fold change relative to the DMSO control. For the positive control, BCBL1 cells were treated with DMSO and chemically reactivated with valproic acid for 48h. Data represent mean ± SEM of two biological replicates, each performed in triplicate, with the exception of the positive control, which is one biological replicate performed in triplicate. (B) Western blot for NEK2 and KSHV ORF45 in PEL cells with intact NEK2 expression (NTC) or depleted NEK2 expression (NEK2 siRNA). GAPDH was used as the loading control. Data are representative of two independent biological replicates. (C) Fold change in gene expression of KSHV lytic genes in PEL cells with intact NEK2 expression (NTC) or depleted NEK2 expression (NEK2 siRNA). Data were normalized to actin and plotted as fold change relative to the NTC. For the positive control, NTC-treated BCBL1 cells were chemically reactivated with valproic acid for 48h. Data represent mean ± SEM of two biological replicates, each performed in triplicate, with the exception of the positive control, which is one biological replicate performed in triplicate. [file crc-23-0430-s02.docx]

**
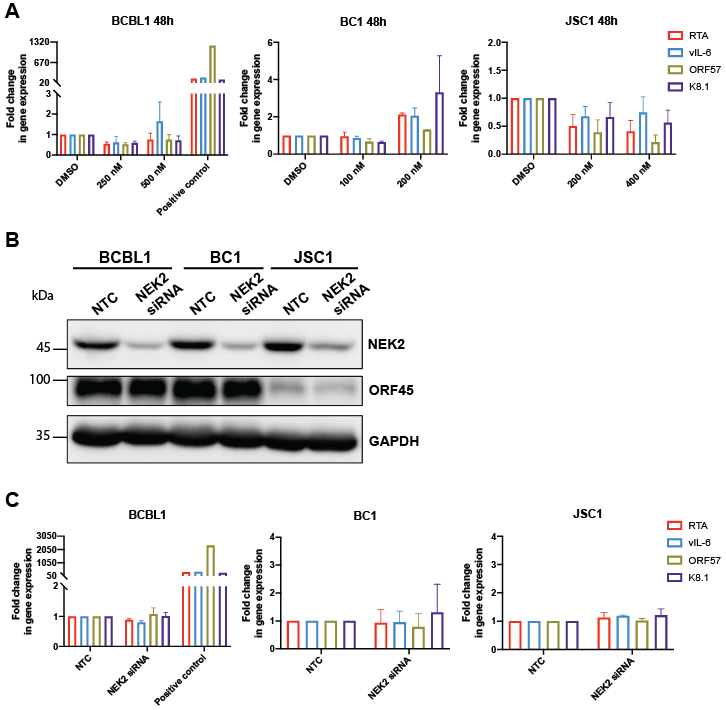
**

**Figure S2. NEK2 inhibition does not induce reactivation of KSHV.** (A) Fold change in gene expression of KSHV lytic genes in PEL cells treated with or without JH295 for 48h. Data were normalized to actin and plotted as fold change relative to the DMSO control. For the positive control, BCBL1 cells were treated with DMSO and chemically reactivated with valproic acid for 48h. Data represent mean ± SEM of two biological replicates, each performed in triplicate, with the exception of the positive control, which is one biological replicate performed in triplicate. (B) Western blot for NEK2 and KSHV ORF45 in PEL cells with intact NEK2 expression (NTC) or depleted NEK2 expression (NEK2 siRNA). GAPDH was used as the loading control. Data are representative of two independent biological replicates. (C) Fold change in gene expression of KSHV lytic genes in PEL cells with intact NEK2 expression (NTC) or depleted NEK2 expression (NEK2 siRNA). Data were normalized to actin and plotted as fold change relative to the NTC. For the positive control, NTC-treated BCBL1 cells were chemically reactivated with valproic acid for 48h. Data represent mean ± SEM of two biological replicates, each performed in triplicate, with the exception of the positive control, which is one biological replicate performed in triplicate.
