# Supplementary material for: Inhibition of NEK2 Promotes Chemosensitivity and Reduces KSHV-positive Primary Effusion Lymphoma Burden
Source: Cancer Res Commun. 2024 Apr 9;4(4):1024–40. doi: 10.1158/2767-9764.CRC-23-0430 (PMC11003453; doi:10.1158/2767-9764.CRC-23-0430)
Supplement: Supplementary Figure 3 — Figure S3. Effects of the Hec1-NEK2 inhibitor, T-1101 tosylate, on PEL viability. (A-C) Viability of PEL cells treated with vehicle control (DMSO, black line) or various concentrations of T-1101 tosylate over time. Data were normalized to the 0h luminescence values for each treatment and represent mean ± SEM. Curves were fitted using non-linear regression and represent three independent biological replicates, each performed in triplicate. (D) IC50 curves of PEL cells treated with various concentrations of T-1101 tosylate or JH295 for 72h and normalized to the DMSO control values. Individual data points are plotted for two-three independent biological replicates, each performed in triplicate. Curves were fitted using non-linear regression and are connected by means. See also Table S3. (E) Viability of PEL cells treated with DMSO, 500 nM T-1101 tosylate, or 500 nM JH295. Data are plotted as individual data points of three independent biological replicates performed in triplicate, normalized to the DMSO control values, and represent mean ± SEM. Data were analyzed using two-way ANOVA with Šídák’s multiple comparisons. ****p < 0.0001. The DMSO data in both graphs in (E) are the same. [file crc-23-0430-s03.docx]

**Figure S3.** **Effects of the Hec1-NEK2 inhibitor, T-1101 tosylate, on PEL viability.** (A-C) Viability of PEL cells treated with vehicle control (DMSO, black line) or various concentrations of T-1101 tosylate over time. Data were normalized to the 0h luminescence values for each treatment and represent mean ± SEM. Curves were fitted using non-linear regression and represent three independent biological replicates, each performed in triplicate. (D) IC50 curves of PEL cells treated with various concentrations of T-1101 tosylate or JH295 for 72h and normalized to the DMSO control values. Individual data points are plotted for two-three independent biological replicates, each performed in triplicate. Curves were fitted using non-linear regression and are connected by means. See also Table S3. (E) Viability of PEL cells treated with DMSO, 500 nM T-1101 tosylate, or 500 nM JH295. Data are plotted as individual data points of three independent biological replicates performed in triplicate, normalized to the DMSO control values, and represent mean ± SEM. Data were analyzed using two-way ANOVA with Šídák’s multiple comparisons. ****p < 0.0001. The DMSO data in both graphs in (E) are the same.
