# Supplementary material for: Inhibition of NEK2 Promotes Chemosensitivity and Reduces KSHV-positive Primary Effusion Lymphoma Burden
Source: Cancer Res Commun. 2024 Apr 9;4(4):1024–40. doi: 10.1158/2767-9764.CRC-23-0430 (PMC11003453; doi:10.1158/2767-9764.CRC-23-0430)
Supplement: Supplementary Figure 4 — Figure S4. NEK2 inhibition in PEL results in decreased expression of the transcription factor, beta-catenin, and the pro-survival protein, Bcl-xL. (A) Western blot for total beta-catenin, phosphorylated beta-catenin (Ser33/Ser37/Thr41, ~92 kDa band), and Bcl-xL in PEL cells treated with or without JH295 for 48h. GAPDH was used as the loading control. Data are representative of three independent biological replicates. (B) Western blot for phosphorylated beta-catenin (Ser33/Ser37/Thr41) and Bcl-xL in PEL cells with intact NEK2 expression (NTC) or depleted NEK2 expression (shRNA). GAPDH was used as the loading control. Data are representative of two-three independent biological replicates. (C) Viability of PEL cells treated with vehicle control (DMSO) or the Bcl-xL inhibitor, A-1155463, for 48h. Data are plotted as individual values from three-four independent biological replicates and normalized to the DMSO control values for each treatment. Data represent mean ± SD and were analyzed using two-way ANOVA with Dunnett’s multiple comparisons. ****p < 0.0001. [file crc-23-0430-s04.docx]

**
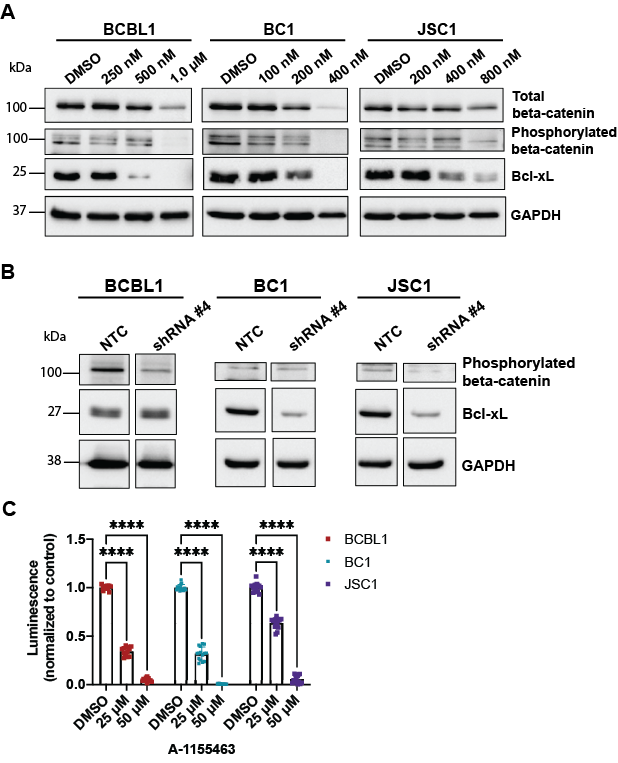
**

**Figure S4. NEK2 inhibition in PEL results in decreased expression of the transcription factor, beta-catenin, and the pro-survival protein, Bcl-xL.** (A) Western blot for total beta-catenin, phosphorylated beta-catenin (Ser33/Ser37/Thr41, ~92 kDa band), and Bcl-xL in PEL cells treated with or without JH295 for 48h. GAPDH was used as the loading control. Data are representative of three independent biological replicates. (B) Western blot for phosphorylated beta-catenin (Ser33/Ser37/Thr41) and Bcl-xL in PEL cells with intact NEK2 expression (NTC) or depleted NEK2 expression (shRNA). GAPDH was used as the loading control. Data are representative of two-three independent biological replicates. (C) Viability of PEL cells treated with vehicle control (DMSO) or the Bcl-xL inhibitor, A-1155463, for 48h. Data are plotted as individual values from three-four independent biological replicates and normalized to the DMSO control values for each treatment. Data represent mean ± SD and were analyzed using two-way ANOVA with Dunnett’s multiple comparisons. ****p < 0.0001.
