# Supplementary material for: Inhibition of NEK2 Promotes Chemosensitivity and Reduces KSHV-positive Primary Effusion Lymphoma Burden
Source: Cancer Res Commun. 2024 Apr 9;4(4):1024–40. doi: 10.1158/2767-9764.CRC-23-0430 (PMC11003453; doi:10.1158/2767-9764.CRC-23-0430)
Supplement: Supplementary Table 1 — Table S1. JH295 IC50 values in PEL cell lines [file crc-23-0430-s05.docx]

| **Table S1.** JH295 IC50 values in PEL cell lines | | | |
| --- | --- | --- | --- |
| *Cell line* | *24h* | *48h* | *72h* |
| BCBL1 | 0.180 µM | 0.164 µM | 0.209 µM |
| BC1 | 0.085 µM | 0.072 µM | 0.077 µM |
| JSC1 | 0.181 µM | 0.129 µM | 0.137 µM |
