# Supplementary material for: Inhibition of NEK2 Promotes Chemosensitivity and Reduces KSHV-positive Primary Effusion Lymphoma Burden
Source: Cancer Res Commun. 2024 Apr 9;4(4):1024–40. doi: 10.1158/2767-9764.CRC-23-0430 (PMC11003453; doi:10.1158/2767-9764.CRC-23-0430)
Supplement: Supplementary Table 2 — Table S2. NCL-00017509 IC50 values in PEL cell lines [file crc-23-0430-s06.docx]

| **Table S2.** NCL-00017509 IC50 values in PEL cell lines | | | |
| --- | --- | --- | --- |
| *Cell line* | *24h* | *48h* | *72h* |
| BCBL1 | 44.29 µM | 23.03 µM | 24.64 µM |
| BC1 | 7.19 µM | 4.11 µM | 3.83 µM |
| JSC1 | 23.55 µM | 6.27 µM | 5.78 µM |
