# Supplementary material for: Inhibition of NEK2 Promotes Chemosensitivity and Reduces KSHV-positive Primary Effusion Lymphoma Burden
Source: Cancer Res Commun. 2024 Apr 9;4(4):1024–40. doi: 10.1158/2767-9764.CRC-23-0430 (PMC11003453; doi:10.1158/2767-9764.CRC-23-0430)
Supplement: Supplementary Table 3 — Table S3. T-1101 tosylate IC50 values in PEL cell lines [file crc-23-0430-s07.docx]

| **Table S3.** T-1101 tosylate IC50 values in PEL cell lines | | | |  |
| --- | --- | --- | --- | --- |
| *Cell line* | *24h* | *48h* | *72h* | *96h* |
| BCBL1 | N.D.^a^ | 0.065 µM | 0.053 µM | 0.031 µM |
| BC1 | N.D. ^a^ | 0.068 µM | 0.047 µM | 0.043 µM |
| JSC1 | N.D. ^a^ | 0.134 µM | 0.137 µM | 0.083 µM |

^a^IC50 value could not be determined
